# Folic-Acid-Conjugated Thermoresponsive Polymeric Particles for Targeted Delivery of 5-Fluorouracil to CRC Cells

**DOI:** 10.3390/ijms24021364

**Published:** 2023-01-10

**Authors:** Sylwia Milewska, Gabriela Siemiaszko, Agnieszka Zofia Wilczewska, Iwona Misztalewska-Turkowicz, Karolina Halina Markiewicz, Dawid Szymczuk, Diana Sawicka, Halina Car, Ryszard Lazny, Katarzyna Niemirowicz-Laskowska

**Affiliations:** 1 Department of Experimental Pharmacology, Medical University of Bialystok, Szpitalna 37, 15-361 Bialystok, Poland; 2 Doctoral School, Medical University of Bialystok, Kilinskiego 1, 15-089 Bialystok, Poland; 3 Faculty of Chemistry, University of Bialystok, Ciolkowskiego 1K, 15-245 Bialystok, Poland; 4 Doctoral School of Exact and Natural Science, University of Bialystok, Ciolkowskiego 1K, 15-245 Bialystok, Poland

**Keywords:** folic acid, colorectal cancer, targeted delivery, drug delivery systems, 5-fluorouracil, folate receptors, mitigator, thermoresponsive polymer, polymeric carriers, RAFT polymerization, PHEA-*b*-PNIPAAm, PNIPAAm, NIPAAm

## Abstract

Colorectal cancer is the fourth most common cancer worldwide and the third most frequently diagnosed form of cancer associated with high mortality rates. Recently, targeted drug delivery systems have been under increasing attention owing to advantages such as high therapeutic effectiveness with a significant depletion in adverse events. In this report, we describe the biocompatible and thermoresponsive FA-conjugated PHEA-*b*-PNIPAAm copolymers as nanocarriers for the delivery of 5-FU. The block copolymers were obtained using RAFT (Reversible Addition–Fragmentation chain Transfer) polymerization and were characterized by methods such as SEC (Size Exclusion Chromatography), NMR (Nuclear Magnetic Resonance), UV–Vis (Ultraviolet–Visible), FT-IR (Fourier Transform Infrared) spectroscopy, and TGA (Thermogravimetric Analysis). Nanoparticles were formed from polymers with and without the drug-5-fluorouracil, which was confirmed using DLS (Dynamic Light Scattering), zeta potential measurements, and TEM (Transmission Electron Microscopy) imaging. The cloud points of the polymers were found to be close to the temperature of the human body. Eventually, polymeric carriers were tested as drug delivery systems for the safety, compatibility, and targeting of colorectal cancer cells (CRC). The biological evaluation indicated high compatibility with the representative host cells. Furthermore, it showed that proposed nanosystems might have therapeutic potential as mitigators for 5-FU-induced monocytopenia, cardiotoxicity, and other chemotherapy-associated disorders. Moreover, results show increased cytotoxicity against cancer cells compared to the drug, including a line with a drug resistance phenotype. Additionally, the ability of synthesized carriers to induce apoptosis and necrosis in treated CRC cells has been confirmed. Undoubtedly, the presented aspects of colorectal cancer therapy promise future solutions to overcome the conventional limitations of current treatment regimens for this type of cancer and to improve the quality of life of the patients.

## 1. Introduction

Current medical statistics indicate that colorectal cancer (CRC) is a widespread disease with a high mortality rate. Rawla et al. reported, based on the GLOBOCAN database, that CRC is the third most deadly and fourth most frequently detected cancer in the world [1]. The analysis of the risk and occurrence of CRC presented by the Polish National Cancer Registry shows that approximately 1 in 23 men and 1 in 25 women will be diagnosed with CRC in their lifetime [2]. The most common treatment combines chemotherapy, surgery, radiotherapy, and targeted therapy [3]. The current chemotherapy involves using 5-fluorouracil (5-FU), an effective drug in various solid tumors, including colorectal, neck, head, and breast cancers [4]. Unfortunately, the problem of resistance to this drug is emerging and much effort has been put into improving 5-FU-based therapy in recent years [5]. One of the methods is to use nanosized drug delivery systems (DDS) that transport the drug to cancer cells and minimize the cytotoxic effect against host cells via restriction of drug penetration and retention in healthy tissues [6]. Compared to a free drug, the drug entrapped in the nanocarrier is protected against enzymatic degradation in the digestive system, allowing the drug to penetrate the bloodstream and reach diseased tissues. To date, the most important known delivery systems for 5-fluorouracil are, e.g., solid lipid, chitosan, poly(lactic-*co*-glycolic acid) (PLGA), silica, polymeric, and folic-acid-conjugated nanoparticles. Their main advantages are enhanced cytotoxic effect and cellular uptake, better targeting efficiency, and prolonged drug release [6,7].

Polymeric therapeutics are an important class of drugs in which the polymer performs an integral role, providing an effective encapsulation, controlled delivery, and drug release [8]. Furthermore, polymers are a class of chemical compounds with advantages such as tunable composition, geometry, size, and ease of derivatization. The latter feature allows attachment to the system ligands for targeting tissues or cells [9]. Drug loading methods include the generation of hydrolytic or acid-sensitive linkage, complexation, and ion pair or permanent bond formation, ensuring the release/action of the drug in the target place [10]. One of the advantages of nanoscale drug carriers is their ability to uptake into tumor sites via the enhanced permeability and retention (EPR) effect. The abovementioned effect, discovered by Maeda et al., is based on a universal pathophysiological mechanism in which macromolecular compounds (above 40 kDa) such as polymeric drug carriers might be accumulated in the tumor area. This in turn leads to achieving targeted delivery and retention of the anticancer agent into tumor tissue. It should be emphasized, that EPR strongly depends on factors associated with the tumor location and vascularization degree as well as the physicochemical properties of the carrier and anticancer agent. In clinical practice, using the EPR effect is the main idea in the passive targeting of solid tumors in cancer nanotherapy [11,12].

It was proved that drug delivery technologies can enhance the health of patients. This is due to minimizing off-target drug accumulation, improving the delivery of a drug to the target site, and facilitating patient compliance [13,14]. Furthermore, the controlled release of the active substance through the use of nanoformulation may be helpful for creating a reduction in adverse events. This can result in fewer medical procedures, lead to lower staff costs, and provide a greater chance of remission [15].

It was established that a plethora of tumors overexpressed folate receptors (FR) on their surface [16]. Therefore, FR-targeting has become the basis of many therapeutic, diagnostic, and imaging methods in the treatment of cancer to enhance cancer cells’ uptake of drug-loaded vehicles. Known strategies of active targeting rely on folic-acid-modified anticancer drugs, antibodies, or antibody–drug conjugates, as well as drug delivery nanoplatforms. Folic acid was used as a targeting ligand because of its low price, availability, non-toxicity, lack of immunogenicity, and presence of functional groups that facilitate its chemical modification without compromising functionality [16,17]. For example, in our previous study, we showed that new polymeric drug carriers, poly(2-hydroxyethylacrylate)-*b*-poly(*N*-vinylcaprolactam) (PHEA-*b*-PNVCL) conjugated with folic acid, used as part of a combinatory treatment, showed improved cytotoxicity of 5-FU against colon cancer cells, while also playing a protective role for healthy tissues [18].

Thermoresponsive polymeric nanocarriers have been intensively studied over the last two decades. They have all of the advantages of drug delivery systems, such as protection against early drug degradation in the body, extended plasma half-life, and increased solubility of non-hydrophilic drugs. Moreover, they are sensitive to external stimuli, allowing for better drug release control. In aqueous solutions, such polymers may exhibit lower or upper critical solution temperature (LCST or UCST). Poly(*N*-isopropylacrylamide) (PNIPAAm) is the most investigated thermoresponsive polymer that was found to have many biomedical applications, including tissue engineering [19,20,21]. As evidence, we recently showed the non-hemolytic nature of polymeric nanoparticles made of polymers containing the cholesterol moiety and PNIPAAm block [22]. Consequently, the biocompatible copolymers consisting of PNIPAAm and poly(2-hydroxyethylacrylate) (PHEA) blocks could provide a high-potential platform for drug delivery [23].

PNIPAAm-based folic-acid-functionalized polymeric drug carriers were demonstrated to be effective in the form of micellar nanoparticles in the delivery of anticancer drugs or model hydrophobic drugs. For instance, Razaei et al. reported that the targeted delivery of paclitaxel via a star-shaped amphiphilic block copolymer (4s[poly(*ɛ*-caprolactone)-*b*-2s(poly(*N*-isopropylacrylamide-*co*-acrylamide)-*b*′-methoxy) poly(ethylene glycol)/poly(ethylene glycol)-folate]) was non-hemolytic and resulted in improved cellular uptake [24,25,26,27,28,29]. Another example is nanocapsules based on PNIPAAm (PNIPAAm-*co*-PMA) and externally functionalized with folic acid, which are promising carriers for doxorubicin [30,31]. Furthermore, magnetic nanoparticles or nanocomposites functionalized with a temperature-sensitive polymer and a folate ligand were tested as doxorubicin delivery systems [32,33,34]. Interestingly, in addition to drug transport, folate-conjugated biopolymer was used to deliver an enzyme that converts a non-toxic prodrug to 5-fluorouracil [35]. Dubé et al. described the synthesis of folate-conjugated copolymer based on NIPAAm and amino-*N*′-ethylenedioxy-bis(ethylacrylamide) [36]. Shin et al. proposed poly(*N*-isopropylacrylamide)-pyrrole-folate nanocomposites as doxorubicin carriers triggered by near-infrared light for chemo-photothermal cancer therapy [37].

Herein we report the synthesis of the series of biocompatible and thermoresponsive folate-conjugated PHEA-*b*-PNIPAAm block copolymers using reversible addition–fragmentation chain transfer (RAFT) polymerization. The preparation of polymers with different ratios of PHEA to PNIPAAm block and different degrees of functionalization with folic acid allows an understanding of the influence of individual components on the formation of micellar structures, the cloud point values, and the strength of interaction with the drug. In consequence, it provides the design of the best drug delivery platform. A comprehensive physicochemical characterization of block copolymers and micelles with encapsulated 5-FU has been carried out. Furthermore, prepared drug carriers were subjected to biological evaluation. Their biocompatibility with representative host cells–red blood cells, monocytic cells, cardiomyocyte cells, skin, and colorectal fibroblast cells was tested. Moreover, cytotoxic activity against three colon cancer cell lines, including those resistant to chemotherapeutic agents, was investigated.

## 2. Results and Discussion

### 2.1. Synthesis and Characterization of PHEA and PHEA-b-PNIPAAm Polymers

**Scheme 1:**
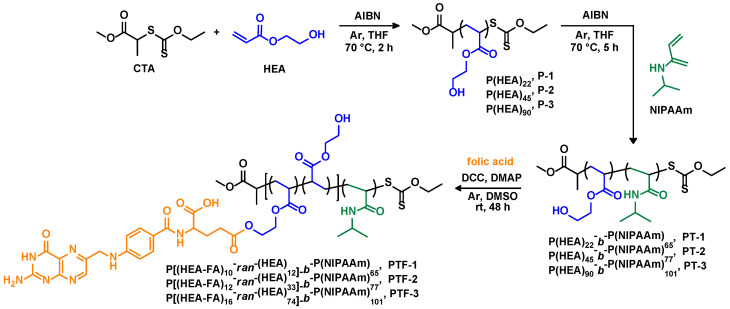
Schematic representation of the synthesis of polymers (considering the theoretical numbers of monomers and folic acid units).

**Figure 1:**
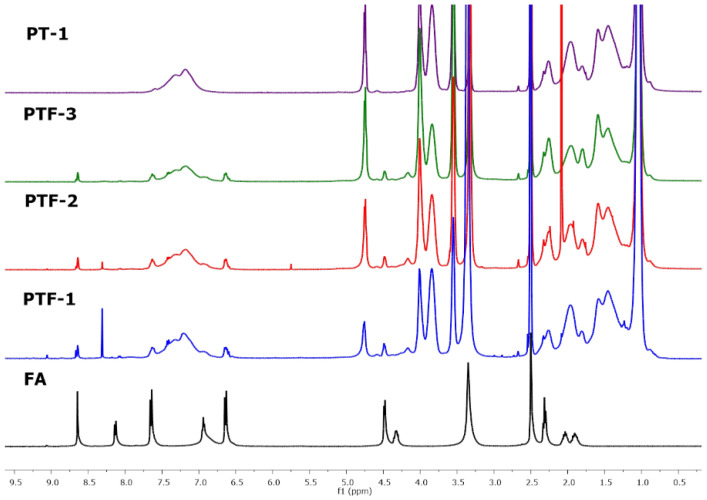
Juxtaposition of ^1^H NMR spectra of folic acid, folate-conjugated **PTF-1**, **PTF-2**, and **PTF-3** polymers, and an example of non-conjugated polymer **PT-1** in DMSO-d_6_.

**Figure 2:**
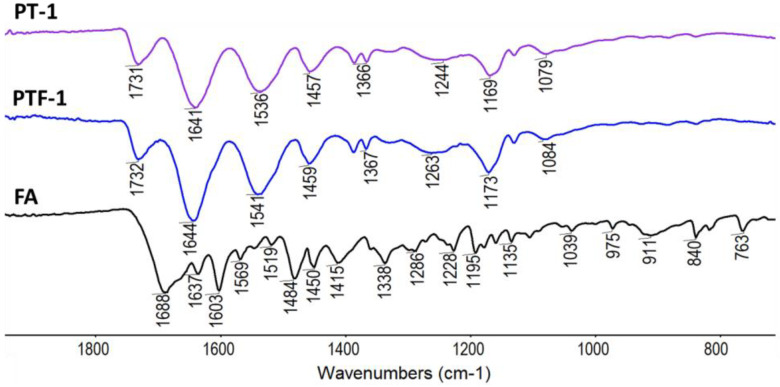
ATR-FT IR spectra of folic acid, **PT-1**, and folic-acid-conjugated **PTF-1** polymer (magnification of the region below 1900 cm^−1^).

PHEA-*b*-PNIPAAm copolymers were obtained from commercially available monomers and simple-chain transfer agent (CTA, methyl 2-((ethoxycarbonothioyl)thio)propanoate) in two steps. First, three poly(2-hydroxyethylacrylate)s (PHEA; P-1, P-2, P-3) of different molecular weights were synthesized by RAFT polymerization using an optimized reaction procedure (Scheme 1, Table S1). Next, chain extensions of each of the PHEA polymers were carried out using RAFT polymerizations of *N*-isopropylacrylamide (NIPAAm) (Scheme 1, Table S2). The obtained thermoresponsive resins (PT-1, PT-2, PT-3) differed in the length of the PHEA block, ensuring a different amount of hydroxyl groups were available for functionalization with folic acid. The polymers and block copolymers were characterized using ^1^H NMR (Table 1, Figure 1 and Figures S1–S3), FT IR (Figure 2 and Figure S4), UV–Vis (Figures S5 and S6), and SEC (Table 1, Figure S7). The results obtained either from NMR or SEC indicate obtaining PHEA homopolymers with molecular weights close to the theoretical ones, growing in a row **P-1**, **P-2**, **P-3** (Table 1, Figure S7). The differences in the polymeric chain lengths were also confirmed using UV–Vis analysis. As shown in Figure S5, the dithiocarbonate groups possess the absorption band at 280 nm [38], where intensity decreases as more monomer is incorporated into the structure of the material. An increase in molecular weight values for block copolymers, in comparison to the corresponding homopolymers, confirms successful RAFT copolymerization. For the copolymer of the lowest theoretical number average molecular weight (M_n,th_) (PT-1), good agreement was observed between M_n,th_ and the value obtained from SEC (Figure S7, Table 1). However, in the cases of **PT-2** and **PT-3**, the M_n,SEC_ values differ from the theoretical ones (Table 1). This is most likely due to chain transfer to solvent, which dictates an upper limit in accessible molecular weight [39,40]. The molecular weights of block copolymers could not be determined from the ^1^H NMR spectra, because the intensity of the dithiocarbonate group signal was at the noise level, making reliable calculations impossible.

### 2.2. Conjugation of Folic Acid

The third step in the synthesis of target polymers was the esterification reaction between the hydroxyl groups of PHEA-*b*-PNIPAAm and the carboxyl group of the folic acid using *N*,*N*′-dicyclohexylcarbodiimide (DCC) and 4-dimethylaminopyridine (DMAP) (Scheme 1, Table S3). We initially performed the coupling of each copolymer with a quantitative amount of folic acid and coupling reagents based on the number of -OH groups, followed by extensive dialysis and freeze-drying. However, the resulting polymers became insoluble in water and phosphate-buffered saline (PBS). Therefore, we carried out the esterification of polymers (PT-(1-3)) with a reduced amount of folic acid, DCC, and DMAP, followed by extensive dialysis leading to the removal of unreacted low molecular weight substrates and by-products. It should be mentioned that two esterification products were probably obtained [41]. We believe that the excess of hydroxyl groups in the system, as well as the appropriate order of adding the reagents, causes only a minimal side reaction between the extreme -NH_2_ group and -COOH groups of the folic acid. The resulting folate-conjugated resins, P[(HEA-FA)-*ran*-(HEA)]-*b*-P(NIPAAm) (PTF-1, PTF-2, PTF-3), had folic acid randomly distributed in the structure of the PHEA block. Furthermore, the resins were assumed to have an equal amount of folic acid but differ in the number of non-functionalized -OH groups, ensuring better solubility in water and PBS.

The successful functionalization with folic acid was supported by ^1^H NMR analysis. The ^1^H NMR spectrum of the PTF-1 polymer shows characteristic signals in the range of 8.7 to 6.5 nm corresponding to the aromatic protons and the pterine moiety of FA (Figure S3). The signal from the amine group overlaps with the strong signal from the -NH group of NIPAAm. A comparison of the spectra of all folic-acid-functionalized polymers with the spectrum of folic acid and the exemplary unfunctionalized polymer PT-1 in DMSO-d_6_ is presented in Figure 1. The presence of folic acid proton signals in the PTF-1, PTF-2, and PTF-3 spectra indicates the successful completion of the coupling reaction.

Furthermore, ATR-FT IR spectroscopy supported the presence of FA in the structure of folate-functionalized polymers. Although signals responding to folic acid or formed ester bonds were overlapped by bands characteristic of functional groups present in the PHEA-b-NIPAAm polymers, some differences may be indicated. Comparing the spectrum showing the enlarged region below 1900 cm^−1^ of the polymer **PT-1** with the spectrum of its folic-acid-conjugated counterpart **PTF-1**, an increase in the signal intensity can be seen at 1644 cm^−1^, corresponding to C=O stretching of the secondary amine in the folic acid structure, compared to the signal at 1732 cm^−1^ (Figure 2) [42]. This is all the more striking because we expected an increase in the intensity of the second signal mentioned due to the C=O stretching of the newly formed ester bond. The same relationship can be seen when comparing polymers in which the theoretical mass content of folic acid is lower than their counterparts, that is, **PTF-2** with **PT-2** and **PTF-3** with **PT-3** (Figure S4).

UV–Vis spectroscopy was used to support the successful post-functionalization of copolymers with folic acid (Figures S5 and S6). The vitamin folate absorbs ultraviolet light and has absorption peaks at 280 and 350 nm [43]. On the spectra of **PTF-1**, **PTF-2**, and **PTF-3** polymers in deionized water in a concentration of 0.2 mg·mL^−1^, these two bands can be observed. However, the signal at 350 nm is more reliable as the signal intensity at 280 nm may be slightly overestimated due to absorption by the dithiocarbonate group (Figure S6). The bands are more intense when more FA moieties are present in the polymer structure, providing an expected series of materials. The absorbance values were used to calculate the exact folic acid content of the samples (Table S6). The calibration curve of folic acid in H_2_O performed previously was applied [18]. The values obtained are equal to 8–10 wt% (Table 1), about three times lower than the theoretical ones. This is due to the incomplete conversion of the reagents in the esterification reaction, but also due to the intensive dialysis through which some of the functionalized PTF chains were removed from the sample.

The molecular weights of folate-conjugated block copolymers were determined using SEC analysis and are given in Table 1 (and Figure S7). After functionalization of PHEA-*b*-PNIPAAm copolymers with FA, there was a M_n,SEC_ and Đ increase (Table 1), which can be explained by the random presence of FA in the polymeric chains.

The thermal stability of the copolymers and their conjugates with FA were studied using thermogravimetric analysis (Figures S8–S10, Table S4). The tested materials decomposed almost totally, showing one significant weight loss between 300–500 °C. The maximum decomposition rate differed depending on the type of polymer. It was around 440 °C for the PHEA homopolymers, 415 °C for the block copolymers, and 405 °C for the FA-conjugated block copolymers (Table S4). The residues observed on the TG curves of the FA-conjugated samples (PTF-1, PTF-2, PTF-3) at 800 °C were larger compared to the ones determined for the block copolymers (PT-1, PT-2, and PT-3) (Table S4). This confirms the presence of FA, which does not decompose totally in an inert atmosphere in the applied temperature range [18]. Additionally, TG analysis was performed for **PTF-1** micelles containing 5-FU (Figure S8, Table S4). In comparison to the **PTF-1** (without 5-FU), a shift of the maximum degradation rate to a lower temperature and an increase in the amount of residue at 800 °C were observed, confirming modification of the material. The glass transition temperature (T_g_) values determined using DSC for PHEA-*b*-PNIPAAm copolymers (82.0, 94.0, 83.2 °C, Table S4) differed from the ones designated for PHEA (<0 °C, Table S4) and known for PNIPAAm homopolymers (>100 °C) [22]. Moreover, the modification of the block copolymers with FA results created a further increase in T_g_ (Table S4). This confirms different compositions of the samples.

### 2.3. Hydrodynamic Diameter and Colloidal Stability Measurements

**Figure 3:**
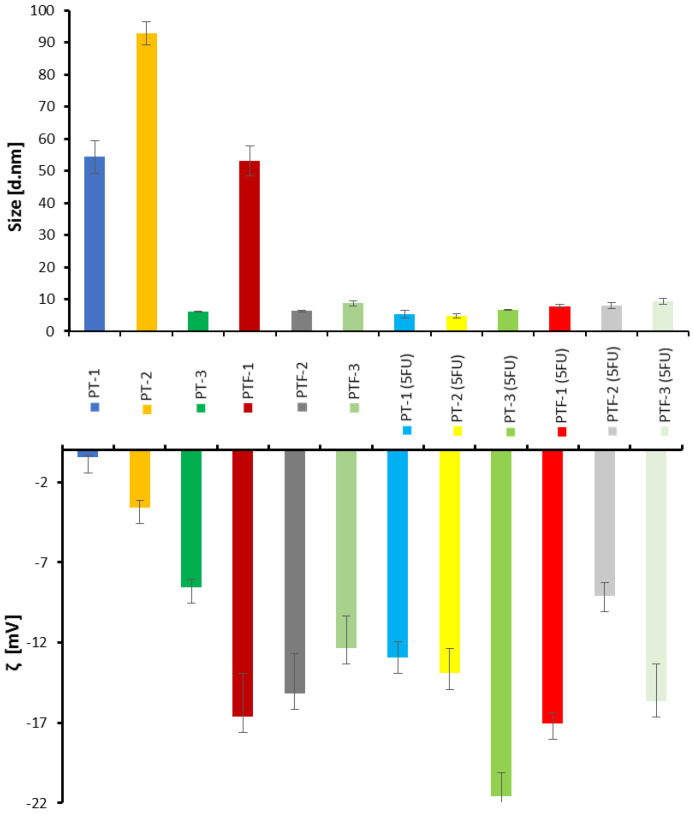
MADLS (size by number) and zeta potential measurements data of all prepared polymers.

Dynamic light scattering (DLS) and zeta potential measurements were used to characterize the polymeric structures formed in aqueous media (Figure 3 and Figure S11). The samples were prepared by dropping a concentrated polymer solution in DMF into the water, followed by dialysis and freeze-drying. The measurements were carried out using solutions with a polymer concentration of 0.5 mg·mL^−1^ after stabilization in the dark for 24 h at 25 °C. The block copolymers **PT-(1-2)** form large unorganized aggregates with hydrodynamic sizes between 40–100 nm, while PT-3, with the longest PHEA chain, forms smaller (around 10 nm) and more stable aggregates. The presence of randomly distributed folic acid molecules in the FA-conjugated PHAE-*b*-NIPAAm chains changes the hydrodynamic sizes of the **PTF-2** but does not significantly affect **PTF-1** and **PTF-3**. However, differences in zeta potential values prove that the presence of folic acid results in the formation of more stable structures (Figure 3, Table 2). Furthermore, all six polymers were analyzed in the presence of the anticancer drug, 5-fluorouracil. The samples were prepared by dropping a mixture of polymer and 5-FU in DMF into the water, followed by dialysis and freeze-drying. In the presence of the drug, all polymers form small structures, below 10 nm. It can be supposed that the interaction between the hydrophilic drug and the hydrophilic block, PHEA, resulted in the formation of smaller and more stable aggregates, which was also confirmed by zeta potential results (Figure 3).

**Figure 4:**
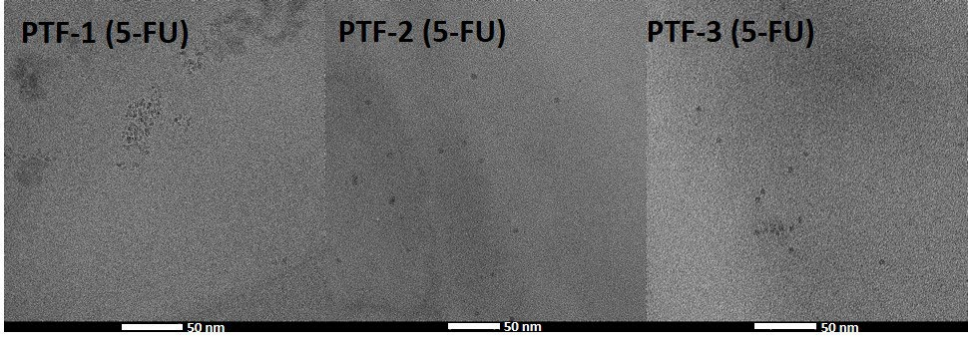
TEM images of polymers with encapsulated 5-fluorouracil.

Transmission electron microscopy (TEM) imaging was performed to determine the morphology of the polymeric structures containing 5-FU in the aqueous solution (Figure 4). All analyzed structures formed spherical shapes with round edges. Polymeric structures with diameters from 5 to 7 nm were observed when polymers with encapsulated drug molecules (PTF-1 (5-FU), PTF-2 (5-FU), and PTF-3 (5-FU)) were imagined. These results correspond with the DLS output. The folic-acid-conjugated polymers **PTF-1** and **PTF-2** during the preparation of TEM samples aggregate to form larger objects (20 nm in PTF-2) or fall apart to form thin polymeric film (PTF-1). Only **PTF-3** polymeric structures were well visible during TEM imaging, and separated structures around 6 nm were observed (see SI, Figure S12). This stability of **PTF-3** may be caused by larger participation of the PHEA block in the polymeric chain in comparison to **PTF-1** and **PTF-2**.

### 2.4. Thermoresponsive Behavior

Turbidimetric measurements were carried out to estimate the cloud point temperature (TCP) of prepared polymers (Table 3). Polymeric particles were dissolved in deionized water at a concentration of 1 mg·mL^−1^ and tested in the temperature range from 28 to 40 °C. The change in the light intensity at 550 nm transmitted through the sample was monitored. The phase transition temperature of copolymers **PT-(1-3)** was approximately 35.5 °C, which is about 3.5 °C more than that of pure PNIPAAm possessing sharp cloud point in the range from 31 °C to 33 °C in water, irrespective of the polymer concentration [44]. This is probably due to the long length of the hydrophilic PHEA block in the PHEA-*b*-PNIPAAm polymers, which causes the solubility despite exceeding the phase transition temperature typical for PNIPAAm (around 32 °C). The appearance of folic acid molecules (hydrophilic) in the copolymer structure increased the T_CP_ of **PTF-1** and **PTF-2** polymers by up to 0.5 °C [45]. This can be explained by the formation of hydrogen bonds through its amino and free carboxylic groups. The phase transition temperature of **PTF-3**, which contained the smallest amount of FA, remained unchanged.

Subsequently, we examined the T_CP_ of structures formed by dissolving polymers in a solution of the drug in DMF, followed by dialysis and freeze-drying. Polymeric carriers with the encapsulated drug were redissolved in deionized water at a concentration of 1 mg·mL^−1^ and tested as particles without a drug. As predicted, the formation of hydrophobic bonds and π-π interactions of the hydrophilic drug molecule with polymers increased their T_CP_. In the case of copolymers **PT-(1-3)**, an increase of approx. 0.5 °C was noted. In turn, polymers conjugated with folate **PTF-(1-3)** showed an increase in the phase transition temperature by 0.5 to 0.9 °C. In conclusion, the designed carriers, together with a specific drug, led to the formation of structures whose T_CP_ was close to the temperature of the human body and higher than that of pure PNIPAAm. Furthermore, superior T_CP_ was achieved compared to some known systems consisting of FA and PNIPAAm [24,29].

### 2.5. Drug Encapsulation and Release

5-Fluorouracil was chosen to investigate the drug loading and controlled release behavior of FA-conjugated thermoresponsive polymers. Drug-loaded micelles were prepared by dissolving the polymer in a DMF solution of 5-FU (to 1 mg 5-FU per 5 mg of drug carrier) and then dropping the solution into vigorously stirred water. Then, dialysis was performed to remove unbound drug and organic solvent, and the content of the dialysis membrane was freeze-dried. Aliquots taken from outside the dialysis membrane were used to indirectly determine the drug loading efficiency (LE) and drug loading content (LC) in the carrier. To increase the drug concentration in the samples, the aliquots were freeze-dried, and the obtained dry residue was redissolved in a small amount of water, followed by HPLC analysis. The encapsulation efficiency calculated on this basis was within the range of 16.8% to 89.4% (Table 4). The yield increased in series from **PT-1** to **PT-3**, in accordance with the growth in the number of hydroxyl and amide groups present in the carrier, indicating the formation of hydrogen bonds between 5-FU and the copolymer. In the case of folic-acid-functionalized copolymers, π-π interactions between FA and the drug may be responsible for the encapsulation of 5-FU. According to this, the LE decreases as the amount of FA in the polymer decreases in the series from **PTF-1** to **PTF-3**. Hydrogen bonds seem to play a minor role, perhaps by obscuring the small units possessing -OH and -NH groups with large FA molecules. The obtained LE values translate directly into drug loading content (LC). They range from 3.3% to 15.2%, with 15.2% being the highest for the compounds with the highest LE, thus 15.2% for **PT-3** and 12.2% for **PTF-1** (Table 4). These are satisfactory results, as most drug carriers are characterized by a low drug content, generally not exceeding 10% [46].

Subsequently, we studied the release of the drug from the 5-FU-loaded polymeric micelles. Carriers prepared in the same way as in the case of studying the efficiency of drug encapsulation were dissolved in imitating-physiological-fluid phosphate-buffered saline. The solution was dialyzed against PBS thermostated at 37 °C for 24 h. The release temperature corresponded to the temperature of the human body in a low-grade fever state. It was aimed at exceeding the T_CP_ of carriers, thus precipitating the polymers and pushing 5-FU out of the polymer chains. Aliquots were taken from outside the dialysis membrane after specified periods of time, and loss was replenished with fresh PBS. The samples were freeze-dried and dissolved in deionized water for further analysis using HPLC. Unfortunately, the calculated drug content was generally hovering around zero. Only in the case of the polymer with a high drug content **PTF-1** was it possible to determine the total release of the adsorbed 5-FU over 24 h as equal to 4.0% (Table 4). Additionally, the influence of the media on drug release was investigated using two selected polymeric systems: **PT-3** and **PTF-1**. In both cases, the degrees of drug release in water could be determined, but they did not exceed 2.0% (Table 4). It can be concluded that in the tested system, the drug is permanently attached to the polymeric structure. Drug loading methods based on complexation or permanent bond formation have already been used in drug delivery. Some of the systems representing these methods are at the stage of clinical trials [14].

### 2.6. Biological Studies

Colorectal cancer (CRC) is a disease that is classified as one of the most common cancers globally [5]. CRC is also one of the most significant contributors to high cancer-related mortality rates [47]. In the treatment of colorectal cancer, both in monotherapy and in combination therapy, it is recommended that the first-line therapy is based on 5-fluorouracil (5-FU). In the past, several regimen modulation strategies, including the introduction of 5-FU-based combination schemes, have been developed and tested to improve the anticancer effectiveness and overcome the clinical drug resistance of 5-FU and 5-FU prodrugs [48]. Unfortunately, the 5-year survival rate of patients with early diagnosed CRC (stages I and II) is just over 60%, and well over 50% of patients are diagnosed at stage III or later when distant metastases have already developed. In this case, the overall 5-year survival rate decreases to only 10% [47].

Treatment schemes that are 5-FU-based have several disadvantages, such as unpredictable severe toxicity, which is generally associated with personal changes in dihydropyrimidine dehydrogenase (DPD) expression, short biological half-life, and strong side effects such as cardiotoxicity or myelosuppression. The aim of developing novel drug delivery systems is to overcome all of these limitations. The DDS can facilitate the slow and sustained release of 5-FU. This further prevents its in vivo degradation and reduces its toxicity. Undoubtedly, the advantages mentioned above would significantly improve the length and quality of life of patients with CRC [49].

To develop effective nanosized drug delivery systems, understanding the interaction between nanoparticles and host cells is a crucial step in biological studies [50]. Consequently, the lack of compatibility at this research step might be a considerable limitation in further clinical introduction [51]. To evaluate whether the encapsulation of 5-FU in PHEA-*b*-PNIPAAm-based carriers would prevent toxic effects on host cells (including human RBC, monocytic cells, skin, colon fibroblast cells, and cardiomyocyte cells), hemolysis assay and cytotoxicity evaluation have been performed.

**Figure 5:**
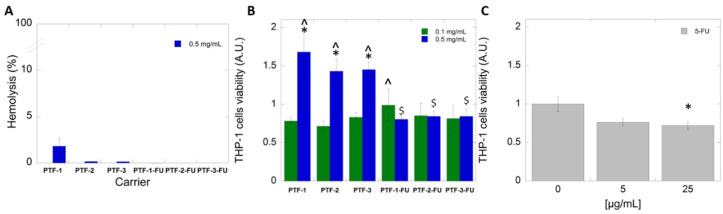
Hemocompatibility of folate-conjugated polymers. Hemolytic activity (panel (**A**)) and viability of monocytic THP-1 cells after the addition of bare folate-conjugated polymers (PTF 1-3) and folate-conjugated polymers with loaded 5-FU (PTF-1-3-FU) (panel (**B**)), and 5-FU (panel (**C**)). Statistical significance for the bare folate-conjugated polymers or folate-conjugated polymers loaded with 5-FU or 5-FU at free form vs. control was marked with (*); comparison of 5-FU concentration at free form vs. polymeric form with or without 5-FU was marked with (^); and comparison of bare folate-conjugated polymers (PTF 1-3) vs. folate-conjugated polymers loaded with 5-FU (PTF-1-3-FU) was marked with ($). *p* ≤ 0.05. The data presented constitute average results from three measurements ± SD.

The first step of the study was focused on the interaction of human RBC with polymeric nanosystems applied at the highest concentration (Figure 5A). For this purpose, the hemolytic activity of developed micelles in free form and loaded with 5-FU was examined. Results showed that both forms of tested carriers (PTF and PTF-FU) did not damage the RBC membrane after 1 h incubation. Consequently, synthesized nanosystems proposed as a carrier for 5-FU meet the criteria foreseen in the pharmaceutical recommendation, where the hemolysis level is classified below 10% [52]. Furthermore, they also conform to the conditions for blood-contacting materials, where the acceptable percentage of hemolysis is below 2% [53]. In effect, proposed carriers might be considered in the further step of the research, including in in vivo evaluation.

During chemotherapy, many cancer patients experience hematological side effects following 5-FU treatment. 5-FU causes severe myelosuppression, leading to morbidity and mortality in cancer patients. This is due to an immunocompromised state and the possibility of serious infection development [54]. As an effect of 5-FU treatment, significant hematologic toxicity appears. This involves a decrease in all types of peripheral blood cell levels, manifested as neutropenia, monocytopenia, thrombocytopenia, and thrombocytosis. In our study, we focus on evaluating the protective effects of synthesized polymeric particles as mitigators against 5-FU-induced hematologic toxicity. For this purpose, THP-1 cells were tested. They are a suitable model of human monocyte/macrophage cells which reflects their physiological function [55]. The choice of these types of cells is also essential because early monocytopenia after chemotherapy is an important risk factor for neutropenia [56]. Our results showed that after treatment with 5-FU, the monocytic cell exerted a ~30–40% reduction in metabolic activity, which is directly associated with a depletion in proliferation and cell viability (Figure 5C). In turn, after the addition of polymeric micelles to the THP-1 cells, an increased ability with regards to proliferation was observed (Figure 5B). Most importantly, the treatment of representative cells by synthesized micelles after loading 5-FU does not significantly affect their metabolic activity and proliferation, independent of applied concentration. This indicates that the favorable influence of the carriers alleviates the cytotoxic effect of antimetabolite.

It is generally accepted that anticancer drugs are powerful tools for individual or combinatory treatment [57,58]. Their application significantly increases survival and depletes the recurrence rate of cancer. However, their use might be limited by cardiotoxicity. During the therapy, cardiotoxicity can be manifested at an early or late stage of treatment. Importantly, cardiac dysfunction might appear as subtle changes in cardiac structure and function up to irreversible heart failure, which is dangerous to the lives of patients [59]. The search for new methods that could restrict the occurrence of cardiac dysfunction is a challenge for current chemotherapeutic strategies.

**Figure 6:**
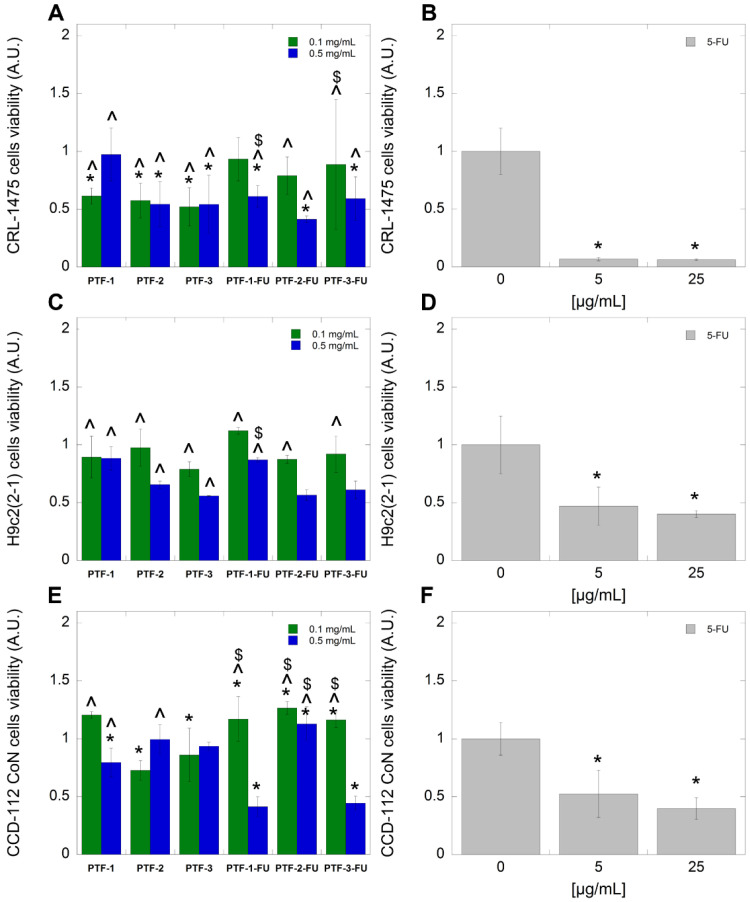
Compatibility of folate-conjugated polymers against representatives of host cells. Viability of cardiomyocyte cells (Panels (**A**,**B**)), skin fibroblast cells (Panels (**C**,**D**)), and colon fibroblast cells (panels (**E**,**F**)) after the addition of bare folate-conjugated polymers (PTF-1-3), folate-conjugated polymers with loaded 5-FU (PTF 1-3-FU), and 5-FU (5 and 25 µg·mL^−1^). Statistical significance for the bare folate-conjugated polymers or folate-conjugated polymers loaded with 5-FU or 5-FU at free form vs. control was marked with (*); comparison of 5-FU concentration at free form vs. polymeric form with or without 5-FU was marked with (^); and comparison of bare folate-conjugated polymers (PTF 1-3) vs. folate-conjugated polymers loaded with 5-FU (PTF-1-3-FU) was marked with ($), *p* ≤ 0.05. The data presented constitute average results from three measurements ± SD.

Results presented in Figure 6A indicate that empty and loaded-with-5-FU polymeric micelles applied at both tested concentrations did not decrease the viability of cardiomyocytes if compared to the control. However, the treatment of cells with 5-FU in free form causes a statistically marked depletion in cell viability—more than 60% (Figure 6B). It should be emphasized that our 5-FU-loaded carriers exert a statistically significant lower cytotoxic effect than 5-FU applied in free form. This indicates the potential of these carriers to prevent the development of chemotherapy-induced cardiotoxicity in cancer patients.

5-Fluorouracil is a potent inhibitor of the proliferation of fibroblasts, the extracellular matrix cells responsible for conferring strength and resiliency of the skin. The patients undergoing 5-FU-based chemotherapy indicated problems with normal wound healing [60]. This is associated with the fact that cytostatic agents, in a non-specific manner, inhibit the proliferation of normal cells, which are responsible for the regeneration process, and exert a negative impact on the wound healing process. Additionally, the adverse effect of 5-FU chemotherapy is recurrent and causes chronic intestinal mucositis. This is also related to the increased production of pro-inflammatory cytokines and the suppression of efficient healing abilities of the mucosa [61]. In our study, we examined the effect of FA-modified micellar nanocarriers as a delivery system of 5-FU on the viability of skin and colon fibroblast cells (Figure 6C–F). Results showed that the treatment of fibroblast cells with 5-FU applied in free form caused a significant reduction in viable cells below 10% in the case of skin fibroblast and ~40% in the case of colon fibroblast (Figure 6D,F). However, using the synthesized carriers with FA moiety resulted in a statistically significant increased viability of treated cells, simultaneously causing a depletion in 5-FU cytotoxicity. In the case of skin fibroblast, the viability was 5-fold higher when cells had been treated with a micellar form of 5-FU. In turn, the treatment of colon fibroblast caused a 2-fold increase in cells’ viability after treatment using folic-acid-modified micelles with loaded 5-FU at concentration 0.1 mg·mL^−1^. The obtained results suggest that the use of a micellar carrier for cytostatic delivery could be helpful in reducing the side and toxic effects related to anticancer treatment.

**Figure 7:**
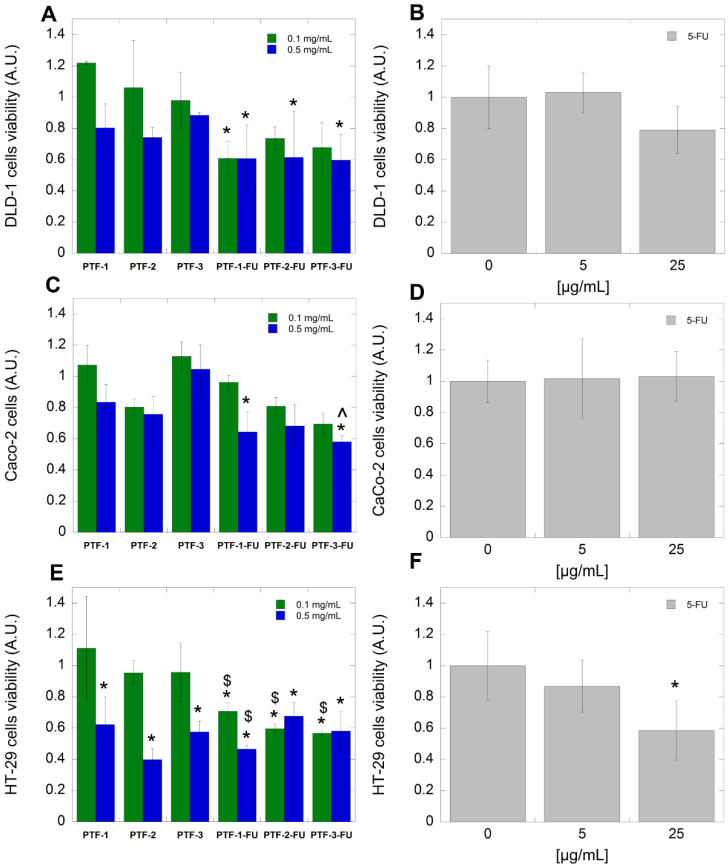
Cytotoxic effect of folate-conjugated polymers against CRC cells. Viability of DLD-1 (panels (**A**,**B**)), CaCo-2 (panels (**C**,**D**)), and HT-29 (panels (**E**,**F**)) cells after the addition of bare folate-conjugated polymers (PTF 1-3), folate-conjugated polymers loaded with 5-FU (PTF-1-3-FU), and 5-FU. Statistical significance for the bare folate-conjugated polymers and folate-conjugated polymers loaded with 5-FU or 5-FU at free form vs. control was marked with (*); comparison of 5-FU concentration at free form vs. polymeric form with or without 5-FU was marked with (^), and comparison of bare folate-conjugated polymers (PTF 1-3) vs. folate-conjugated polymers loaded with 5-FU (PTF-1-3-FU) was marked with ($), *p* ≤ 0.05. The data presented constitute average results from three measurements ± SD.

In the last step of the research, the anticancer potential of synthesized polymeric particles as drug carriers of 5-FU against three different CRC cell lines was investigated. For this purpose, DLD-1, CaCo-2, and HT-29 were chosen for testing sensitivity to 5-FU applied in free form and incorporated form (Figure 7A–F). Tested cells were incubated with empty and loaded nanoparticles at two concentrations (0.1 and 0.5 mg·mL^−1^) and with 5-FU added in the free form at two concentrations (5 and 25 µg·mL^−1^) for 24 h. As shown in Figure 7B,D,F, treatment of all kinds of cancerous cell lines with 5-FU in the free form resulted in a lack of statistically significant depletion in survival if the drug was applied at a concentration of 5 µg·mL^−1^; at the highest tested dose of 5-FU, i.e., 25 µg·mL^−1^, the cell viability of DLD-1 (Figure 7B) and HT-29 (Figure 7F) cells was reduced to 80% and 60%, respectively.

Interestingly, the viability of CaCo-2 cells in the presence of 5-FU has remained unchanged, independent of the applied concentration (Figure 7D). However, the incubation of CRC cells with synthesized carriers indicated divergent effects (Figure 7A,C,E). In the case of DLD-1 cells and CaCo-2 cells, known in the literature as being resistant to 5-FU, our results showed that encapsulation of 5-FU inside the synthesized carriers caused a statistically significant depletion in cell growth and viability by 40% in comparison to the untreated control. The increase in the cytotoxic effect of 5-FU added in the encapsulated form was also observed in the case of HT-29 cells, known as intermediately sensitive to 5-FU. In effect, it could be concluded that evaluated cancer cells were considerably more sensitive to particle-mediated treatment than the free drug.

**Figure 8:**
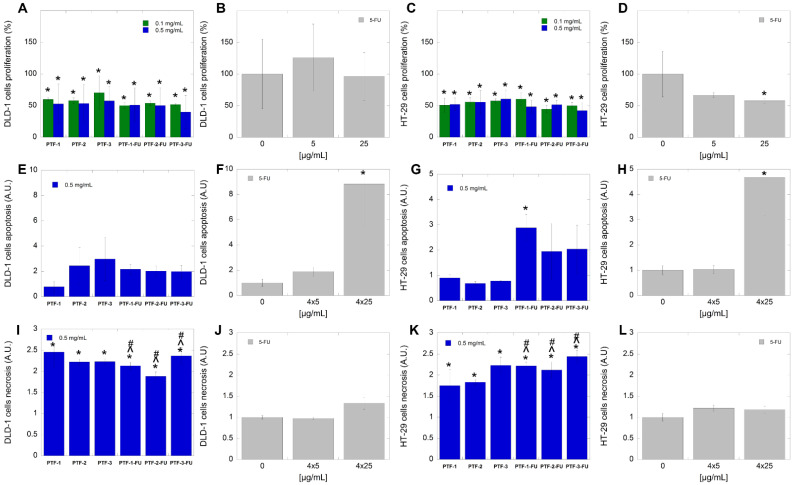
Inhibition of proliferation and mode of action of folate-conjugated polymers against CRC cells. Proliferation of DLD-1 (panels (**A**,**B**)) and HT-29 (panels (**C**,**D**)) cells after the addition of bare folate-conjugated polymers (PTF 1-3), folate-conjugated polymers loaded with 5-FU (PTF-1-3-FU) and 5-FU. Induction of apoptosis in DLD-1 (panels (**E**,**F**)) and HT-29 (panels (**G**,**H**)) cells after the addition of bare folate-conjugated polymers (PTF 1-3), folate-conjugated polymers loaded with 5-FU (PTF-1-3-FU), and 5-FU. Induction of necrosis in DLD-1 (panels (**I**,**J**)) and HT-29 (panels (**K**,**L**)) cells after the addition of bare folate-conjugated polymers (PTF 1-3), folate-conjugated polymers loaded with 5-FU (PTF-1-3-FU), and 5-FU. Statistical significance for the bare folate-conjugated polymers or folate-conjugated polymers loaded with 5-FU or 5-FU at free form vs. control was marked with (*); comparison of 5-FU concentration (5 or 5 × 4 µg·mL^−1^) at free form vs. polymeric form with or without 5-FU was marked with (^); and comparison of 5-FU concentration (25 or 4 × 25 µg·mL^−1^) at free form vs. polymeric form with or without 5-FU was marked with (#). *p* ≤ 0.05. The data presented constitute average results from three measurements ± SD.

It is established that the major mechanisms responsible for the development of a 5-FU resistance phenotype in cancer cells are associated with impaired drug uptake and target alterations. Based on antimetabolites, the mode of action induces cytotoxicity via interfering with the biosynthesis of nucleic acids RNA and DNA. We suggested that the application of synthesized FA-functionalized polymeric particles might lead to overcoming 5-FU resistance [62]. The abovementioned suggestion could be accomplished via interaction with FA receptors which provide drug uptake and then inhibition of proliferation, and finally induction of cell death. In order to better explain the proposed mode of action, two CRC cell lines of DLD-1 and HT-29 cells have been engaged [63,64]. The chosen CRC cells possess different molecular and genetic profiles including RER status and TGFbIIR mutations, as well as the expression level of the folate receptors [65]. To evaluate the impact of the synthesized carriers on cancer cells’ metabolic activity and proliferation, a resazurin assay has been performed. Results presented in Figure 8A–D indicate that FA-functionalized polymeric particles significantly inhibit cell division if compared to the control. In the case of DLD-1 cells, after treatment with 5-FU-loaded carriers, more than 50% of cells possessed markedly decreased metabolic activity, while the treatment of cells with 5-FU at free form indicated a lack of impact on cell proliferation. In turn, in the case of HT-29 cells, similar activity has been observed for both forms of the drug (free and encapsulated). It should be emphasized that in both treated cell’s lines, an inhibition of cell proliferation has been noted after the treatment of cells with empty carriers, which suggests that some cytotoxic modes of action might be characteristic for carriers alone. In another set of experiments, to elucidate the mode of action, bioluminescent- and fluorometric-based assays were used. After 24 h exposition of CRC cells to synthesized carriers, a bioluminescent assay that measures the exposure of phosphatidylserine (PS) on the outer leaflet of the cell membrane during the apoptotic process was performed. Results indicated that, in the case of DLD-1 cells, loaded carriers caused apoptosis at a level similar to 5-FU applied at a concentration of 20 µg·mL^−1^at free form. In turn, the treatment of HT-29 cells with 5-FU-loaded polymeric carriers caused a 2-fold increased apoptosis if compared to drugs applied at free form in a concentration of 20 µg·mL^−1^. To investigate more accurately the molecular mechanism involved in observed killing activity, we investigated whether the necrosis pathway might be involved in this process. For this purpose, a fluorescent-based technique was applied. Upon the loss of membrane integrity, the dye enters the cell, then binds to DNA, and generates a fluorescent signal. As demonstrated in Figure 8I–L, synthesized carriers, both empty and loaded with 5-FU, are able to induce cell death more effectively than a drug in free form, independent of applied concentration (20 or 100 µg·mL^−1^). This suggests that the main mechanism of action of the proposed carriers is the induction of necrosis in treated cells. The abovementioned suggestion might explain the cytotoxic effect (decreased viability and inhibition of proliferation) observed for empty carriers.

Using 5-FU (or its derivatives) encapsulated in polymeric carriers to treat CRC has been reported recently. Moodley and Singh [66] synthesized polymeric mesoporous silica nanoparticles (MSNs) functionalized with biocompatible polymers, chitosan, and poly(ethylene glycol) for the delivery of 5-FU. The results showed better controlled release profiles (15–65%) over 72 h and cell-specific cytotoxicity against cancer cells. After the in vitro assessment, they also found that these formulations are safe and efficient delivery systems with great potential for in vivo applications. Öztürk et al. [67] have examined the 5-fluorouracil-loaded PCL nanoparticles. The obtained formulations showed high encapsulation efficiency of about 93%. Cytotoxicity studies revealed that PCL nanoparticles containing 5-FU exhibited a higher antiproliferative effect than free form 5-FU on the Caco-2 cell line. In turn, Wang et al. [68] designed PLGA nanoparticles conjugated with folic acid. They found lower LC_50_ for encapsulated 5-FU against HT-29 cancer cells if compared to the free form of the drug. The authors indicated that the presence of the folic acid moiety on the surface of the nanoparticles is responsible for the rapid uptake of the nanoparticle into the cell. Those mentioned above and the currently presented results agree with our previously published study. We introduced the synthesis and biological activity of well-defined polymeric drug delivery systems based on PHEA-PNVCL with folic acid moiety. These studies showed that appropriately created carriers could be used in 5-fluorouracil complexation and combinatory treatment. The statement was established based on cytotoxic results, in which a significant decrease in the viability of the representatives of CRC cells has been noted [18].

## 3. Experimental Section

### 3.1. Materials

The initiator, 2,2′-azobis(2-methylpropionitrile) (AIBN, 98%, MERCK, Darmstadt, Germany) was recrystallized from methanol. Monomer, *N*-isopropylacrylamide (NIPAAm, 99%, ACROS, Geel, Belgium) was recrystallized from toluene–hexane (60:40, *v/v*), before use. 2-Hydroxyethyl acrylate (96%, Aldrich, Burlington, MA, USA), folic acid (FA, pure, Alfa Aesar, Kandel, Germany), *N,N*′-dicyclohexylcarbodiimide (DCC, 98%, Fluorochem, Pune, India), 4-dimethylaminopyridine (DMAP, ≥99%, Aldrich), 5-fluorouracil (5-FU, Ebewe Pharma, Unterach am Attersee, Austria), pyrene (Py, ≥99%, Aldrich), phosphate-buffered solution (PBS, pH = 7.4, GIBCO, Monza, Italy), and Dulbecco’s Modified Eagle Medium (DMEM, GIBCO) were used as received. Potassium *O*-ethylcarbonodithioate [21] and methyl 2-((ethoxycarbonothioyl)thio)propanoate [69] were synthesized according to previously reported procedures. Organic solvents THF, EtOH, and DMF were purchased from Avantor Performance Materials, Poland, S.A., DMSO from Chempur, and MeOH for HPLC from Merck. DMSO and THF were dried over activated molecular sieves 4 Å and stored under argon. The deuterated solvent was purchased from Armar Chemicals (DMSO-d_6_). For all experiments, glassware was dried in a laboratory oven at 120 °C for 20 h.

### 3.2. Methods

#### 3.2.1. Nuclear Magnetic Resonance (NMR)

1H NMR spectra were recorded on a Bruker Avance II 400 spectrometer at 400 MHz. Chemical shifts δ are given in ppm, referenced to the solvent peak of CDCl_3_, defined at δ = 7.26, or DMSO-d_6_, defined at δ = 2.50.

#### 3.2.2. Attenuated Total Reflectance Fourier Transform Infrared Spectroscopy (ATR-FTIR)

ATR-FTIR spectra were taken on a Thermo Scientific Nicolet 6700 FTIR spectrophotometer, possessing an ATR accessory. Spectra were collected in the wavenumber range from 4000 to 500 cm^−1^ by adding 64 scans with a resolution of 4 cm^−1^.

#### 3.2.3. Size Exclusion Chromatography (SEC)

The polymers, copolymers, and their conjugates with folic acid were characterized using size exclusion chromatography (SEC). DMF/LiBr (10 mM) solution was used as eluent at 55 °C with a flow rate of 1.0 mL min^−1^. Prior to injections, the samples were dissolved in the eluent to a concentration of 5 mg mL^−1^ and filtered through 0.45 μm PTFE filters. The samples were analyzed using two column sets, KF-804L and KF-805L Shodex (homopolymers), or TSKgel Alpha-2500 and Alpha-3000 Tosh Bioscience (block copolymers and their FA conjugates), coupled with a three-detector system: a refractometer thermostated at 35 °C (Optilab Rex, Wyatt technology, Santa Barbara, CA, USA), a UV detector (Prostar, Varian, Palo Alto, CA, USA) set at 254 nm, and a multi-angle laser light scattering (MALS) detector (Mini Dawn, 3 angles, Wyatt technology). The dn/dc of PHEA (0.076 mL·g^−1^) was taken from the literature [70]. The dn/dc value of block copolymers and their FA conjugates was assumed to be equal to one of the PNIPAAm homopolymers (0.087 mL·g^−1^) [71].

#### 3.2.4. Dynamic Light Scattering (DLS) and ζ Potential

The colloidal stability of the polymeric systems was examined using Zetasizer Ultra (Malvern Panalytical Ltd., Malvern, UK) equipped with a 10 mW helium/neon laser (l = 633 nm) at 25 °C. The instrument settings were optimized automatically using the ZS XPLORER software (Malvern Panalytical Ltd., Malvern, UK). All measurements were carried out using a Multiangle Dynamic Light Scattering (MADLS) detection system. The measurements of polymer samples (prepared according to procedures given in Section 3.3.4 and Section 3.3.5) with conc. 0.5 mg·mL^−1^ in reversible osmosis water were completed at 25 °C after stabilization in the dark for 24 h. The analyses were repeated five times, two extreme results were rejected, and the remaining mean of three results was taken.

#### 3.2.5. Ultraviolet–Visible Spectroscopy (UV–Vis)

UV–Vis spectra were collected using a Jasco V-670 Spectrophotometer at a wavelength range of 250–460 nm. PHEA-*b*-PNIPAAm polymers and folate-conjugated PHEA-*b*-PNIPAAm polymers in conc. 0.2 mg·mL^−1^ in deionized water at 25 °C were analyzed.

#### 3.2.6. Turbidimetry

Thermo-regulated UV–Vis spectroscopy was applied to determine the cloud points of the samples. Particle suspensions were prepared using deionized water at a concentration of 1 mg·mL^−1^. A Jasco V-670 Spectrophotometer was used to record spectra at a wavelength of 550 nm in the absorbance mode with a heating rate of 0.5 °C per minute in the temperature range of 28–40 °C. The temperature at which optical transmittance was starting to drop sharply was considered T_CP_.

#### 3.2.7. Thermogravimetric Analysis (TGA)

Thermogravimetric analyses were conducted using a Mettler Toledo Star TGA/DSC unit. Polymeric samples weighing 2–3 mg placed in aluminum oxide crucibles were heated from 50 °C to 900 °C. The heating rate was equal to 10 K·min^−1^, and the argon flow rate was 40 mL·min^−1^. An empty pan was used as a reference.

#### 3.2.8. Differential Scanning Calorimetry (DSC)

Differential scanning calorimetry (DSC) was performed using a Mettler Toledo Star DSC unit. A sample weighing 2–3 mg was placed in an aluminum crucible and sealed. A sample was first heated from 25 to 200 °C at a rate of 10 °C·min^−1^, held isothermally for 5 min, and then cooled to −50 °C at a rate of −20 °C·min^−1^. Two heating/cooling cycles under an argon flow rate of 200 mL·min^−1^ were performed, and an empty pan was used as a reference. The glass transition temperature (T_g_) was taken as the midpoint of the heat capacity change in the second heating run.

#### 3.2.9. Transmission Electron Microscope

In order to be analyzed with a Transmission Electron Microscope (TEM), plunge-freeze-drying was applied to prepare samples. Briefly, solutions of polymers (pretreated according to Section 3.3.4 and Section 3.3.5) with a concentration of 0.1 mg·mL^−1^ in deionized water were prepared. Next, 3 µL of the sample was placed on a holey carbon copper grid (200 mesh copper, SPI Supplies, West Chester, PA, USA) and excess of the solution was taken away with a tissue; this was repeated twice. Treated grids were frozen with liquid nitrogen (LN2) and dried overnight on a vacuum pump. A Tecnai G2 X-Twin microscope was used to take images that were taken at the accelerating 200 kV voltage applying LN2 cryotrap for the microscope column.

#### 3.2.10. Freeze-Drying

Samples were freeze-dried on Christ Alpha 1-2 LDplus apparatus equipped with a double chamber. Solutions of polymers in distilled water were frozen with liquid N_2_, followed by freeze-drying under 0.013 mbar pressure for 24 h.

#### 3.2.11. Dialysis

Dialysis of folate-conjugated polymers was performed against DMSO or distilled water using dialysis membrane Spectra/Por^®^ 6 (MWCO 1000, surface width 18 mm) at 25 °C. Dialysis of polymeric micelles was performed against distilled water using dialysis membrane ZelluTrans/ROTH T1 (MWCO 3500, surface width 46 mm) at 25 °C.

### 3.3. Synthetic Procedures

#### 3.3.1. General Procedure for RAFT Polymerization of HEA (P-(1-3))

Methyl 2-((ethoxycarbonothioyl)thio)propanoate (CTA) and 2-hydroxyethyl acrylate (HEA) were dissolved in dry THF under argon. The mixture was immersed in an oil bath and thermostated at 70 °C while stirring. AIBN as a solution in dry THF was added, and polymerization proceeded for 2 h at 70 °C until monomer conversion was high (96–98%). After cooling, the polymer was isolated and purified via precipitation in cold Et_2_O, collected, and dried under reduced pressure, affording the product (PHEA, P-(1-3)) as a colorless sticky resin. The HEA unit numbers and molecular weights (M_n,NMR_) were calculated by integrating the ^1^H NMR signals of -CH_2_CH_3_ and -CH_2_CH_2_OH protons and are given in Table S1. **P-1**: ^1^H NMR (DMSO-d_6_, 400 MHz): δ 4.80–4.70 (m, OH), 4.61 (q, J = 7.2 Hz, 2H, -CH_2_CH_3_), 4.05–3.90 (m, -CH_2_CH_2_OH), 3.60–3.50 (m, -CH_2_CH_2_OH), 2.45–2.15 (m, -CH_2_CHC(O)-), 1.90–1.40 (m, -CH_2_CHC(O)-), 1.37–1.31 (m, 3H, -CHCH_3_), 1.08–1.03 (m, 3H, -CH_2_CH_3_). FTIR (neat): 3396 (OH), 2949, 2882, 1724 (C=O), 1448, 1394, 1331, 1238, 1159, 1073, 1022, 889, 842, 757 cm^−1^.

#### 3.3.2. General Procedure for RAFT Polymerization of PHEA with NIPAAm (PT-(1-3))

Polymer PHEA and *N*-isopropylacrylamide were dissolved in anhydrous THF under argon. The mixture was immersed in an oil bath and thermostated at 70 °C while stirring. AIBN as a solution in anhydrous THF was added, and polymerization proceeded for 5 h at 70 °C. After cooling, the polymer was isolated and purified via double precipitation in cold Et_2_O, collected, and dried under reduced pressure, affording the product as (PHEA-*b*-PNIPAAm, PT-(1-3)) a white powder. **PT-1**: ^1^H NMR (DMSO-d_6_, 400 MHz): δ 6.90–7.60 (m, -NH-), 4.85–4.70 (m, OH), 4.10–3.95 (m, -OCH_2_CH_2_OH), 3.95–3.70 (m, -CH(CH_3_)_2_), 3.60–3.50 (m, -OCH_2_CH_2_OH), 2.40–2.15 (m, -CH_2_CHC(O)O-), 2.15–1.15 (m, -CH_2_CHC(O)O-, -CH_2_CHC(O)NH-), 1.15–0.85 (m, CH(CH_3_)_2_). FTIR (neat): 3282 (OH), 3075, 2970, 2933, 2875, 1732 (C=O), 1641, 1536, 1457, 1386, 1366, 1329, 1244, 1169, 1130, 1079 cm^−1^.

#### 3.3.3. General Procedure for Conjugation of PHEA-*b*-PNIPAAm with Folic Acid (PTF-(1-3))

Polymer PHEA-*b*-PNIPAAm was freeze-dried right before the reaction. Folic acid was dissolved in anhydrous DMSO under argon for several hours. The folic acid solution was added to the polymer, DCC, and DMAP, and the reaction proceeded for 48 h at room temperature under argon. Then, the reaction mixture was filtered through a cotton pad and dialyzed against DMSO for 48 h and subsequently against water for 96 h. The aqueous solution of the polymer was freeze-dried, affording the product (PTF-(1-3)) as a yellow light solid. **PTF-1**: ^1^H NMR (DMSO-d_6_, 400 MHz) characteristic signals: δ 8.68–8.58 (m, Ar-H, FA), 7.68–7.58 (m, 2 x -Ar-H, FA), 7.55–6.75 (m, -NHCH(CH_3_)_2_), 6.68–6.56 (m, 2 x -Ar-H, FA), 4.85–4.65 (m, OH), 4.52–4.43 (-CH_2_NH-, FA), 4.10–3.95 (m, -OCH_2_CH_2_OH, -OCH_2_CH_2_OFA), 3.95–3.70 (m, -CH(CH_3_)_2_), 3.60–3.50 (m, -OCH_2_CH_2_OH), 2.40–2.15 (m, -CH_2_CHC(O)O-), 2.15–1.15 (m, -CH_2_CHC(O)O-, -CH_2_CHC(O)NH-), 1.15–0.90 (m, CH(CH_3_)_2_). FTIR (neat): 3291 (OH), 2971, 2933, 2874, 1732 (C=O), 1644, 1541, 1458, 1387, 1367, 1263, 1171, 1130, 1080 cm^−1^.

#### 3.3.4. Polymeric Micelles Formation

15 mg of the polymer were dissolved in 1.5 mL of DMF and added dropwise to 15 mL of distilled water with constant stirring. Next, the sample was dialyzed against 1 L of distilled water for 24 h, changing the water in which the membrane was immersed twice (after 1 h and 3 h). Finally, the polymeric micelles solution (content of the membrane) was freeze-dried and stored in the dark.

#### 3.3.5. Drug Encapsulation

Drug-encapsulated polymer particles were formed as follows: 15 mg of the polymer was dissolved in 1.5 mL solution of 5-FU in DMF (c = 2 mg·mL^−1^) and added dropwise to 15 mL of distilled water with constant stirring. Next, it was dialyzed against 1 L of distilled water for 24 h, changing the water twice (after 1 h and 3 h). The membrane content (polymeric particles with encapsulated drug) was freeze-dried and further analyzed.

The encapsulation efficiency was measured indirectly by measuring the content of the non-encapsulated drug for each sample as follows: aliquots outside the membrane (30 mL) from each 1 L of water used in dialysis were taken, combined (90 mL in total), and freeze-dried. Next, samples were dissolved in 3 mL of deionized water, filtered using 0.45 µm PTFE filters, and analyzed using high-performance liquid chromatography (HPLC). First, a calibration curve of 5-FU (1.0–0.01 mg·mL^−1^) aqueous solutions was made using HPLC with a UV detector. A ThermoScientific Hypersilil GOLD 25005-254630 RP column (4.6 × 250 mm, 5 μm) was used for separation, maintaining the column temperature at 20 °C. The standards and samples were analyzed using a mobile phase of water and methanol (90:10, *v/v*) at a flow rate of 1.0 mg·mL^−1^. The volume of injection was 20 μL. The peak of 5-FU was detected at the wavelength of 265 nm.

Drug loading efficiency (LE) and drug loading content (LC) were calculated as follows:

$$\mathrm{LE}=\frac{\mathrm{mass}\;\mathrm{of}\;5\mathrm{FU}\;\mathrm{in}\;\mathrm{carriers}}{\mathrm{mass}\;\mathrm{of}\;5\mathrm{FU}\;\mathrm{feed}}$$

$$\mathrm{LC}=\frac{\mathrm{mass}\;\mathrm{of}\;5\mathrm{FU}\;\mathrm{in}\;\mathrm{carriers}}{\mathrm{mass}\;\mathrm{of}\;5\mathrm{FU}\;\mathrm{loaded}\;\mathrm{carriers}}$$

#### 3.3.6. Drug Release

5 mg of polymeric micelles with the encapsulated drug were dissolved in 5 mL of PBS and dialyzed against 100 mL of PBS thermostated at 37 °C while stirring. Aliquots (10 mL) were taken from outside the membrane after 15 min, 30 min, 45 min, 60 min, 90 min, 2 h, 4 h, 6 h, 8 h, and 24 h while adding PBS each time to maintain the total PBS volume of 100 mL. Aliquots were freeze-dried, dissolved in 1 mL of deionized water, and further analyzed using HPLC. First, a calibration curve of 5-FU solutions in PBS (0.1–0.001 mg·mL^−1^) was made using HPLC with a UV detector. The same conditions and RP column were used for drug release analysis, as for drug encapsulation. As a result, the drug content in the samples oscillated at around zero. For this reason, only the highest value for all samples after 24 h, calculated without the effect of dilution, is given in Table 4. Drug release from selected samples (PT-3 and PTF-1) was also performed in distilled water using an analogous procedure to investigate the effect of the medium on 5-fluorouracil released from micelles.

### 3.4. Biological Studies

#### 3.4.1. Hemolysis Assay

The hemolytic activity was tested using human red blood cells (RBCs) suspended in phosphate-buffered saline (PBS) (hematocrit ~5%) with a high applied concentration of empty or 5-FU-entrapped polymeric agents −0.5 mg·mL^−1^. Briefly, the RBCs were incubated with tested agents for 1 h at 37 °C. After the incubation, samples were centrifuged, and absorbance was measured at 540 nm. Hemolysis was calculated according to the following equation: Hemolysis (%) = [(As − An)/(Ap − An) × 100)], where Ap, As, and An are the absorbance of the positive control, test sample, and negative control, respectively. The positive control was the RBCs lysed with 1% Triton X-100, and the negative control was the human red blood cell suspension treated with PBS.

#### 3.4.2. Cell Culture

Human colorectal adenocarcinoma cell lines (DLD-1, CaCo-2, and HT-29), human colorectal fibroblasts (CCD-112CoN), skin fibroblasts (CRL-1475), human monocytic cell line (THP-1), and rat embryonic cardiomyocytes (H9c2(2-1)) were obtained from the American Type Culture Collection (ATCC). The DLD-1, CaCo-2, CCD-112 CoN, and CRL-1475 cells were grown in RPMI 1640 medium, line HT-29 in McCoy’s 5a medium supplemented with 10% fetal bovine serum (FBS) and 1% penicillin/streptomycin, at 37 °C and 5% CO_2_. The THP-1 cells were grown in RPMI 1640 medium supplemented with 10% heat-inactivated FBS. The 1% penicillin/streptomycin and 2-mercaptoethanol were added at 37 °C in a 5% CO_2_ atmosphere, so the final concentration was 0.05 mM. The H9c2(2-1) cells were grown in Dulbecco’s modified Eagle’s Medium supplemented with 10% FBS. 1% penicillin/streptomycin was added, and cells were cultured at 37 °C in 5% CO_2_-air.

#### 3.4.3. Cytotoxicity Assay

In vitro cytotoxicity was evaluated by performing neutral red assay of the representatives of the healthy host (cardiomyocyte, skin fibroblast, and colon fibroblast cells) and colorectal cancer cells. In brief, polymeric carriers in free form or encapsulated with 5-FU form at concentrations of 0.1 and 0.5 mg·mL^−1^ were added to treated cells and incubated for 24 h. Simultaneously, the cells were treated with 5-FU applied at concentrations of 5 and 25 µg·mL^−1^. After exposure, the percentage of viable cells was measured using spectrophotometric methods. For this purpose,10 µL of 0.33% neutral red solution was added to each well and incubated. Following this, neutral red was removed after 2–3 h, and then to the cells, neutral red assay fixative (100 μL) was thoroughly added. As a final step, the fixative solution was removed, and the incorporated dye was then solubilized in an adequate volume of solubilization solution (100 μL). After that, the absorbance was measured at 540 nm, and the results were normalized to the control.

In another set of experiments the proliferation and metabolic activity of representatives of CRC cells–DLD-1 and HT-29 cells were determined using resazurin-based assay. For this purpose, the cells were treated with the tested polymeric carriers, 5-FU-loaded polymeric carriers, and free 5-FU, and applied at concentrations of 5 and 25 µg·mL^−1^. After 24 h of exposure, 10 μL of resazurin reagent was added to each well. Then, the cells were incubated for 2 h in the dark at 37 °C in a 5% CO_2_ atmosphere. Absorbance was recorded at 570 nm using a microplate reader. The results were normalized to the control.

During the next series of experiments, the viability and metabolic activity of THP-1 monocytic cells were evaluated after treatment with the tested polymeric micelles applied as free or encapsulated with 5-FU form using the MTS assay. After 24 h of exposure, 20 μL of MTS reagent was added to each well. Then, the cells were incubated for 2 h in the dark at 37 °C in a 5% CO_2_ atmosphere. Absorbance was measured at 490 nm using a microplate reader. The results were normalized to the control.

#### 3.4.4. Mode of Action–Apoptosis and Necrosis Detection

To examine the ability of the synthesized carriers to induce apoptosis and necrosis, multiplex assay including bioluminescent annexin v assay and fluorometric assay–necrosis assay were performed. In brief, polymeric carriers in free form or encapsulated with 5-FU form at concentrations of 0.5 mg·mL^−1^ were added to treated cells and incubated for 24 h. Simultaneously, the cells were treated with 5-FU applied at concentrations 4 × 5 and 4 × 25 µg·mL^−1^. Then, an equal volume (100 µL) of 2× Detection Reagent was added. After 24 h of exposure, luminescence and fluorescence ex 485 nm em 530 nm signal were collected. The results were normalized to the untreated control.

## 4. Statistical Analysis

Statistical analyses were performed using Statistica 13.3 software (StatSoft Inc., Tulsa, OK, USA). Three replicates were measured per time point in each cell experiment. The results were normalized to the control and reported as mean ± standard deviation. For statistical calculations, a one-way analysis of variance (ANOVA) with Dunnett’s correction was used. Statistical significance was accepted at *p* < 0.05.

## 5. Conclusions

Current cancer treatment regimens help to increase survival rates and prognosis, which undoubtedly results in a pharmacoeconomic aspect as a reduction in costs is associated with the treatment process. Despite this, the challenge of fighting against cancer is not over, especially in the case of CRC, considered one of the most aggressive types of cancer.

Created carriers loaded with 5-FU showed significantly lower cytotoxicity against representative host cells and a notable reduction in the viability of CRC cells, including those with a resistance phenotype. Using multiplex-based assay, the ability of synthesized carriers to induce apoptosis and necrosis in treated CRC cells has been confirmed.

The presented polymeric nanosystem may function as a mitigator of 5-FU-induced cytotoxicity against normal cells and a potent drug delivery system against cancer cells. Additionally, it could simplify the therapeutic approaches and overcome the problems of the current 5-FU delivery strategies.

Finally, it could be concluded that the obtained results have underlined the potential of using folate-conjugated polymeric carriers, which could play an essential role in the treatment of colorectal cancer.

## Figures and Tables

**Table 1:** Summary of molecular weights of copolymers based on SEC.

Polymer	wt% FA_th_^a^	wt% FA ^b^	M_n,th_ (g·mol^−1^) ^c^	M_n,NMR_ (g·mol^−1^) ^d^	M_n,SEC_ (g·mol^−1^) ^e^	Ð ^e^
**P-1**	-	-	2710	3500	2740	1.87
**P-2**	-	-	5220	5800	6640	1.55
**P-3**	-	-	10,210	8700	8300	1.97
**PT-1**	-	-	9210	-	9070	1.40
**PT-2**	-	-	12,600	-	9780	1.42
**PT-3**	-	-	19,610	-	11,040	1.55
**PTF-1**	33	10	13,700	-	13,040	1.65
**PTF-2**	29	9	17,840	-	14,700	1.51
**PTF-3**	26	8	26,500	-	17,040	1.57

^a^ wt% FAth = (equivFA × MFA × convFA): Mn,th. ^b^ Calculated using the calibration curve of FA absorbance at 280 nm in distilled H_2_O vs. concentration (Figure S5). ^c^ Mn,th = MCTA + MHEAּ × equivHEA × convHEA + MNIPAAm × equivNIPAAm ּ× convNIPAAm + equivFA ּ× MFA × convFA; numbers of equivalents and degrees of conversion are taken from Tables S1–S3; we assume that the conversion of folic acid is quantitative. ^d^ Measured using ^1^H NMR. ^e^ Measured using SEC-RI-MALS.

**Table 2:** MADLS (size by number) and zeta potential measurement data of all prepared polymers.

Polymer	Parameters	
	Size (nm)	ζ [mV]
**PT-1**	54.33 ± 4.98	−0.44 ± 0.07
**PT-2**	92.83 ± 3.66	−3.56 ± 0.45
**PT-3**	6.21 ± 0.11	−8.56 ± 0.54
**PTF-1**	53.10 ± 4.60	−16.61 ± 2.67
**PTF-2**	6.37 ± 0.28	−15.18 ± 2.51
**PTF-3**	8.69 ± 0.70	−12.34 ± 2.02
**PT-1 (5FU)**	5.33 ± 1.27	−12.94 ± 1.02
**PT-2 (5FU)**	4.73 ± 0.63	−13.91 ± 1.52
**PT-3 (5FU)**	6.66 ± 0.11	−21.55 ± 1.47
**PTF-1 (5FU)**	7.77 ± 0.54	−17.02 ± 0.65
**PTF-2 (5FU)**	8.02 ± 0.91	−9.08 ± 0.84
**PTF-3 (5FU)**	9.4 ± 0.96	−15.64 ± 2.3

**Table 3:** Turbidimetry data for PHEA-*b*-PNIPAAm and P[(HEA-FA)-*ran*-(HEA)]-*b*-PNIPAAm polymers ^a^.

Polymer	T_CP_ [°C]	
	Without 5-FU	With 5-FU
**PT-1**	35.7	36.0
**PT-2**	35.5	36.0
**PT-3**	35.4	36.0
**PTF-1**	36.1	36.5
**PTF-2**	36.0	36.6
**PTF-3**	35.6	36.5

^a^ Solutions of polymeric micelles in water in conc. 1 mg·mL^−1^ in H_2_O at 25 °C.

**Table 4:** Drug encapsulation and release data.

Polymer	LE [%] ^a^	LC [%] ^b^	Release after 24 h [%]	
			PBS	H_2_O
**PT-1**	16.8	3.3	n.d.	-
**PT-2**	29.5	5.6	n.d.	-
**PT-3**	89.4	15.2	n.d.	1.5
**PTF-1**	69.2	12.2	4.0	2.0
**PTF-2**	45.5	8.3	n.d.	-
**PTF-3**	41.7	7.7	n.d.	-

^a^ Drug loading efficiency (LE) was determined using HPLC. ^b^ Drug loading content (LC) was determined using HPLC. n.d.—not detected.

## Data Availability

Not applicable.

## Supplementary Materials

The following supporting information can be downloaded at https://www.mdpi.com/article/10.3390/ijms24021364/s1.

## Abbreviations

5-FU: 5-Fluorouracil; ATCC: American Type Culture Collection; ATR-FTIR: attenuated total reflectance Fourier transform infrared spectroscopy; CP: cloud point; CRC: colorectal cancer; CTA: chain transfer agent; D: dispersity; DCC: N.N′-dicyclohexylcarbodiimide; DDS: drug delivery systems; DLS: dynamic light scattering; DMAP: 4-dimethylaminopyridine; FA: folic acid; FBS: fetal bovine serum; FR: folic receptor; FT-IR: Fourier-transform infrared spectroscopy; HPLC: high-performance liquid chromatography; LC: drug loading content; LCST: lower critical solution temperature; LEE: drug loading efficiency; LN2: liquid nitrogen; Mn: molecular weight; NMR: nuclear magnetic resonance; PBS: phosphate-buffered saline; PHEA: Poly(2-hydroxyethyl acrylate); PHEA-b-PNVCL: Poly(2-hydroxyethylacrylate)-b-poly(N-vinylcaprolactam); PLGA: Poly(lactic-co-glycolic acid); PNIPAAm: Poly(N-Isopropylacrylamide); RAFT/MADIX: reversible addition–fragmentation chain transfer/macromolecular design via the Interchange of Xanthates; RBCs: red blood cells; SEC: size exclusion chromatography; TEM: transmission electron microscope; TGA: thermogravimetric analysis; UCST: upper critical solution temperature; UV–Vis: ultraviolet–visible spectroscopy.
