# Supplementary material for: Folic-Acid-Conjugated Thermoresponsive Polymeric Particles for Targeted Delivery of 5-Fluorouracil to CRC Cells
Source: Int J Mol Sci. 2023 Jan 10;24(2):1364. doi: 10.3390/ijms24021364 (PMC9861804; doi:10.3390/ijms24021364)
Supplement: Supplementary file 1 [file ijms-24-01364-s001.zip › SI_PHEA FA PNIPAAm_14-12-22.pdf]

Folic-Acid-Conjugated Thermoresponsive Polymeric Particles for Targeted Delivery of 5-Fluorouracil to CRC Cells

Sylwia Milewska [1,2,†], Gabriela Siemiaszko [3,†], Agnieszka Zofia Wilczewska [3], Iwona Misztalewska-Turkowicz [3], Karolina Halina Markiewicz [3], Dawid Szymczuk [3,4], Diana Sawicka [1], Halina Car [1], Ryszard Lazny [3] and Katarzyna Niemirowicz-Laskowska [1,*]

[1] Department of Experimental Pharmacology, Medical University of Bialystok, Szpitalna 37, 15-361 Bialystok, Poland
[2] Doctoral School, Medical University of Bialystok, Kilinskiego 1, 15-089 Bialystok, Poland
[3] Faculty of Chemistry, University of Bialystok, Ciolkowskiego 1K, 15-245 Bialystok, Poland
[4] Doctoral School of Exact and Natural Science, University of Bialystok, Ciolkowskiego 1K, 15-245 Bialystok, Poland
* Correspondence: katarzyna.niemirowicz@umb.edu.pl
† These authors contributed equally to this work.

Table of content

Table S1. Details of **P-(1-3)** polymers synthesis. 2

Table S2. Details of **PT-(1-3)** polymers synthesis. 2

Table S3. Details of **PTF-(1-3)** polymers synthesis 2

Table S4. Thermal properties of the polymers 3

Table S5. Calculations of content of folic acid 3

Figure S1. ^{1}H NMR spectrum of exemplary PHEA polymer, **P-1**, in DMSO-*d*$_6$. 4

Figure S2. ^{1}H NMR spectrum of exemplary PHEA-*b*-NIPAAm polymer, **PT-1**, in DMSO-*d*$_6$. 5

Figure S3. ^{1}H NMR spectrum of exemplary P[HEA-FA)-*ran*-(HEA)]-*b*-P(NIPAAm) polymer, **PTF-1**, in DMSO-*d*$_6$. 5

Figure **S4. T**he juxtaposition of ATR-FT IR, and folic acid (magnification of the region below 1900 cm-1) – left, and FT IR (in CHCl3) – right – spectra of PT-2, PTF-2, PT-3, PTF-3 polymers. 5

Figure S5. UV-Vis spectra of PHEA-*b*-PNIPAAm polymers, **PT-(1-3)**, in conc. 0.2 mg·ml^{-1} in deionized water at 25 ˚C. 6

Figure S6. UV-Vis spectra of PTF-1, PTF-2, and PTF-3 polymers in conc. 0.2 mg·mL^{-1} in deionized water at 25 ˚C. 6

Figure S7. Normalized SEC-RI traces (A, C) and SEC-UV traces (B, D) of PHEA, PHEA-*b*-PNIPAAm, and P[(HEA-FA)-*ran*-(HEA)]-*b*-PNIPAAm polymers 6

Figure S8-10. TG curves (top panel) and DTG curves (bottom panel) of **P-3**, **PT-3**, and **PTF-3**. 7-8

Figure S11. MADLS (size by number) measurements data of prepared polymers, PT-(1-3) and PTF-(1-3), with or without presence of 5-FU… 9

Figure S12. TEM images of polymers modified with folic acid 9

Table S1. Details of **P-(1-3)** polymers synthesis.

Product	CTA			HEA			AIBN			THF (mL)	Conv[a] (%)	$M_{n,\ NMR}$ [a] ($g{\cdot}mol^{-1}$)	$M_{n,\ th}$ ($g{\cdot}mol^{-1}$)
	Eq	n (mmol)	m (mg)	Eq	n (mmol)	V (mL)	Eq	n (mmol)	m (mg)				
**P-1**	1.0	2.0	416.6	22.4	44.8	5.15	0.1	0.2	32.8	10.0	96.0	3500.0	2707.5
**P-2**	1.0	1.0	208.3	44.8	44.8	5.15	0.1	0.1	16.4	10.0	98.0	5800.0	5218.6
**P-3**	1.0	0.5	104.2	89.6	44.8	5.15	0.1	0.0	8.2	10.0	97.0	8700.0	10209.2

[a]Determined by ^{1}H NMR.

Table S2. Details of **PT-(1-3)** polymers synthesis.

Product	PHEA				NIPAAm			AIBN			THF (mL)	Conv[a] (%)	$M_{n,\ th}$ ($g{\cdot}mol^{-1}$)
	Substrate	Eq	n (mmol)	m (mg)	Eq	n (mmol)	m (g)	Eq	n (mmol)	m (mg)			
**PT-1**	**P-1**	1.0	0.14	370.8	64.5	8.84	1.0	0.01	0.001	0.16	4.5	89.0	9206.1
**PT-2**	**P-2**	1.0	0.12	600.8	76.8	8.84	1.0	0.01	0.001	0.16	4.5	85.0	12601.9
**PT-3**	**P-3**	1.0	0.09	890.8	101.3	8.84	1.0	0.01	0.001	0.16	4.5	82.0	19607.0

[a]Determined by ^{1}H NMR.

Table S3. Details of **PTF-(1-3)** polymers synthesis.

Product	PHEA-*b*-PNIPAAm				FA			DCC			DMAP			DMSO (mL)	$Conv_{th}$ (%)	$M_{n,th}$ ($g{\cdot}mol^{-1}$)
	Sub-strate	Eq	n (mmol)	m (mg)	Eq	n (mmol)	m (mg)	Eq	n (mmol)	m (mg)	Eq	n (mmol)	m (mg)			
**PTF-1**	**PT-1**	1.0	0.033	300.0	10.2	0.331	146.3	11.1	0.362	74.6	1.1	0.036	4.4	6.0	100.0	13695.5
**PTF-2**	**PT-2**	1.0	0.024	300.0	11.9	0.283	124.7	13.0	0.311	64.1	1.3	0.031	3.8	6.0	100.0	17840.0
**PTF-3**	**PT-3**	1.0	0.015	300.0	15.6	0.239	105.5	17.2	0.263	54.3	1.7	0.026	3.2	6.0	100.0	26502.1

[a]Determined by ^{1}H NMR.

Table S4. Thermal properties of the polymers.

Sample ID	Maximum of the degradation rate (°C)	Residue at 800 °C (%)	T_g[a] (°C)
**P-1**	440	5.1	-2.1
**P-2**	440	4.8	-3.3
**P-3**	440	5.6	-5.3
**PT-1**	415	3.7	82.0
**PT-2**	415	2.8	94.0
**PT-3**	415	4.2	83.2
**PTF-1**	405	5.3	119.1
**PTF-2**	405	7.4	108.2
**PTF-3**	410	7.1	103.5
**PTF-1_5FU**	395	7.9	-
**5FU**	310	5.4	-

[a]Determined by DSC from the second heating run performed with a heating rate of 10 °C·min^{-1}.

Table S5. Calculations of content of folic acid.

	A (y)[a]	$c_{polymer}$ (mg·mL^{-1})	c_{FA} (mg·mL^{-1}) (x)[b]	%wt (%)[c]
**PTF-1**	1.04	0.2	0.0193	9.65
**PTF-2**	1	0.2	0.0185	9.23
**PTF-3**	0.84	0.2	0.0151	7.56

[a] Maximum absorbance taken from UV-Vis spectra of polymers in conc. $c_{polymer}$ in deionized water at 25 °C.

[b] Concentration of folic acid calculated from calibration curve of folic acid in H_2O (y = 0.048 x + 0.114).

[c] %wt = ($c_{polymer}$: c_{FA}) · 100%

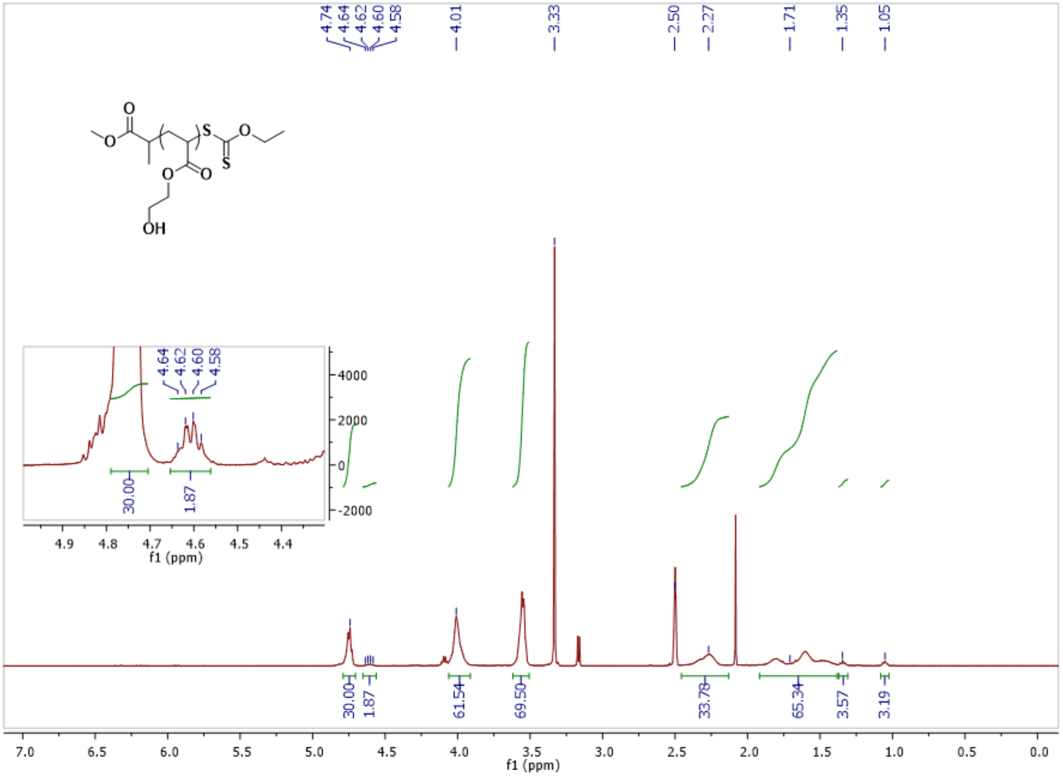

Figure S1. ^{1}H NMR spectrum of exemplary PHEA polymer, **P-1**, in DMSO-d_6.

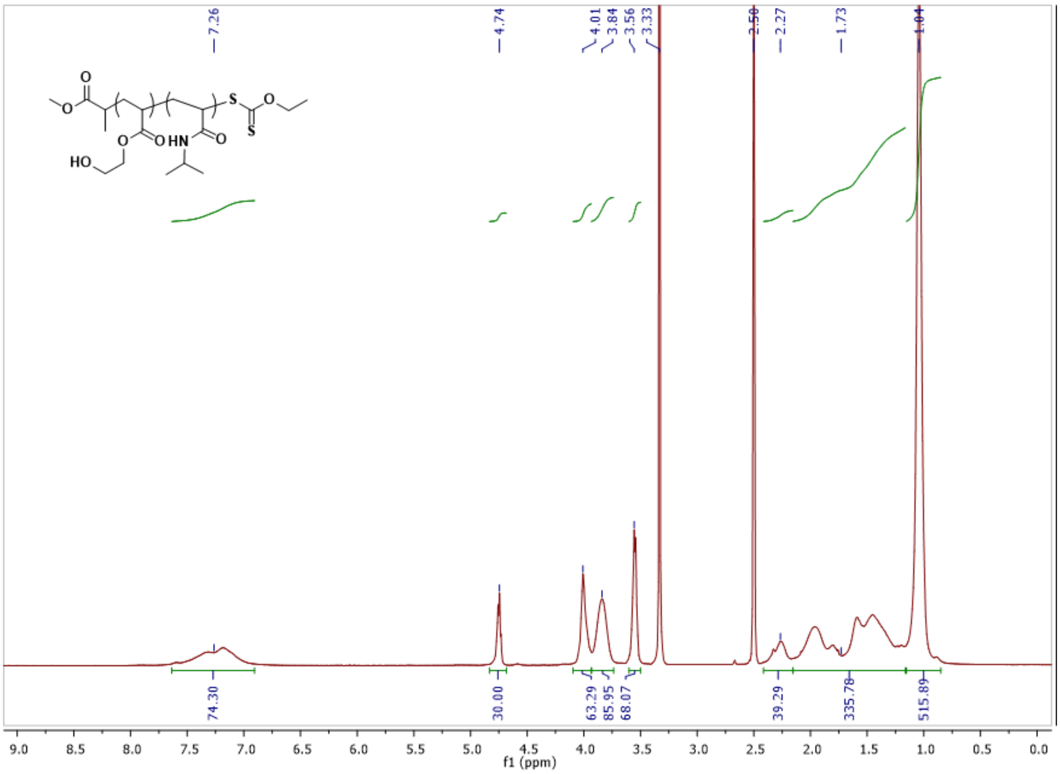

Figure S2. ^{1}H NMR spectrum of exemplary PHEA-*b*-PNIPAAm polymer, **PT-1**, in DMSO-d_6.

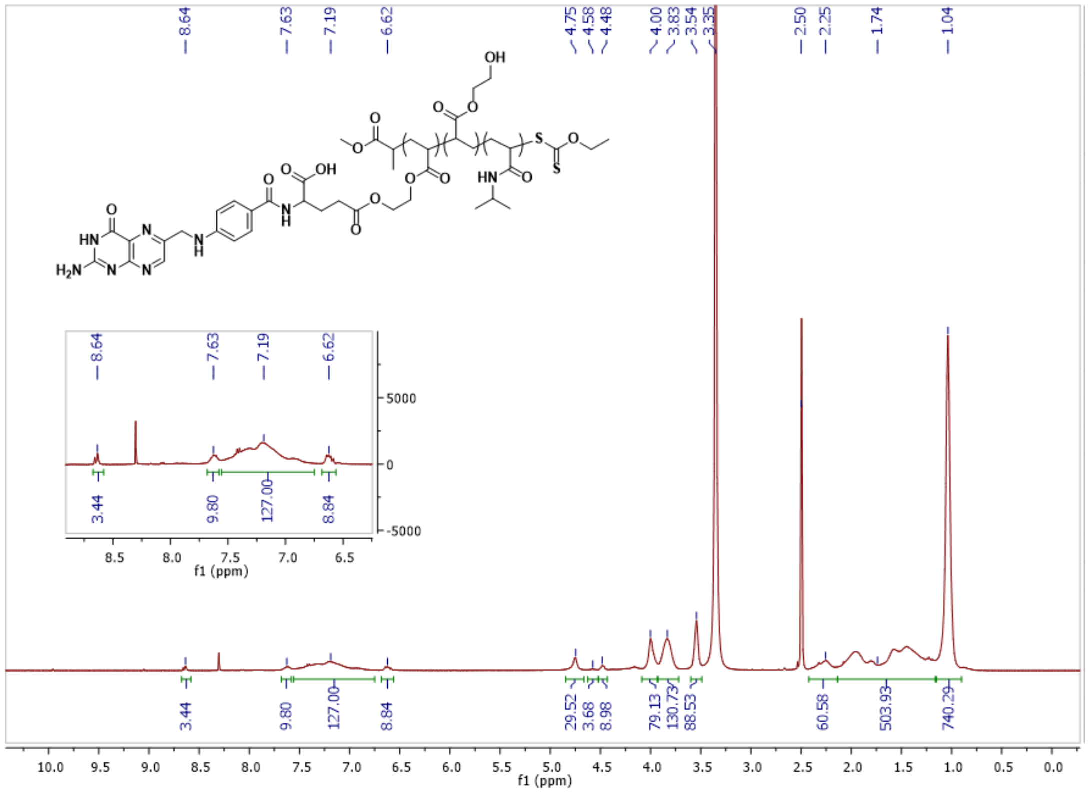

Figure S3. ^{1}H NMR spectrum of exemplary P[HEA-FA)-*ran*-(HEA))-*b*-P(NIPAAm) polymer, **PTF-1**, in DMSO-d_6.

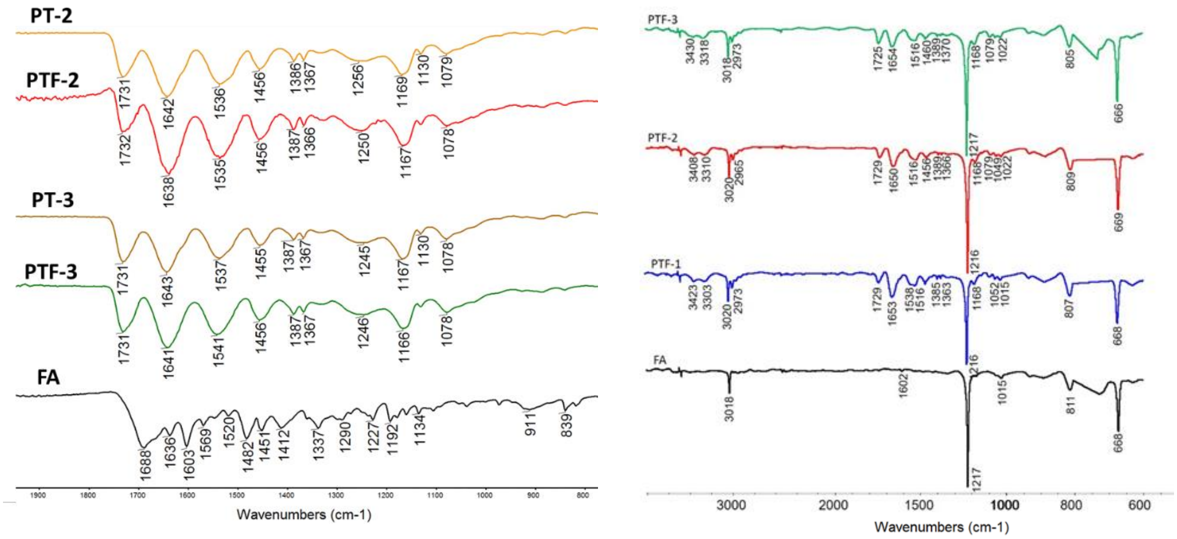

Figure S4. The juxtaposition of ATR-FT IR, and folic acid (magnification of the region below 1900 cm^{-1}) – left, and FT IR (in $CHCl_3$) – right – spectra of **PT-2**, **PTF-2**, **PT-3**, **PTF-3** polymers.

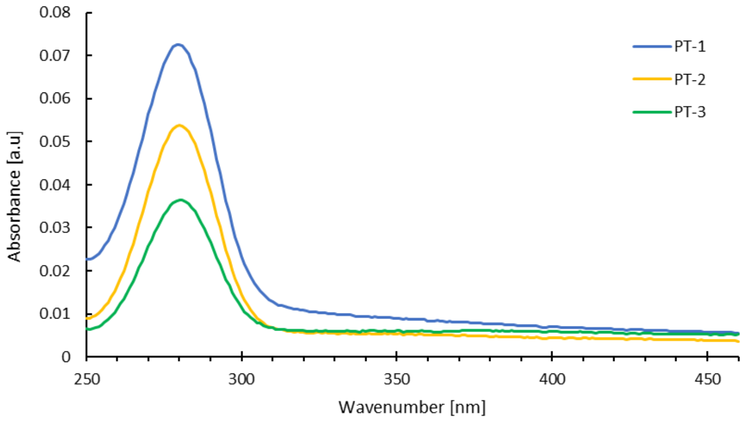

Figure S5. UV-Vis spectra of PHEA-*b*-PNIPAAm polymers, **PT-(1-3)**, in conc. 0.2 mg·ml^{-1} in deionized water at 25 °C.

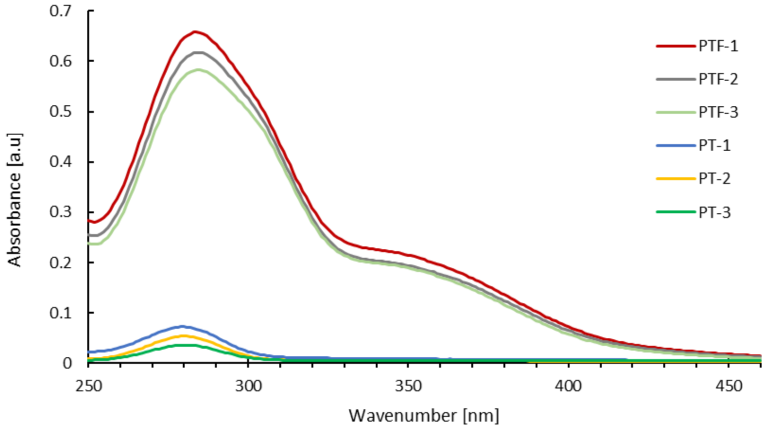

Figure S6. UV-Vis spectra of PT-1, PT-2, PT-3, PTF-1, PTF-2, and PTF-3 polymers in conc. 0.2 mg·mL^{-1} in deionized water at 25 °C.

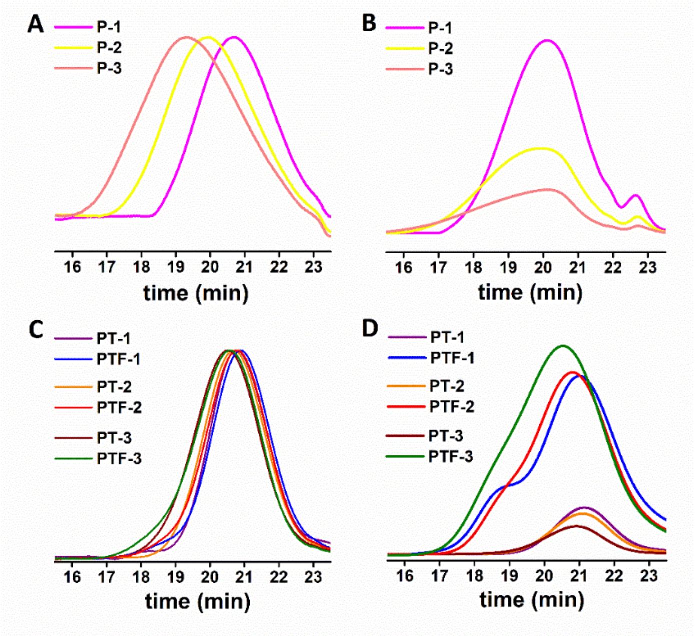

Figure S7. Normalized SEC-RI traces (A, C) and SEC-UV traces (B, D) of PHEA, PHEA-*b*-PNIPAAm, and P[(HEA-FA)-*ran*-(HEA)]-*b*-PNIPAAm polymers.

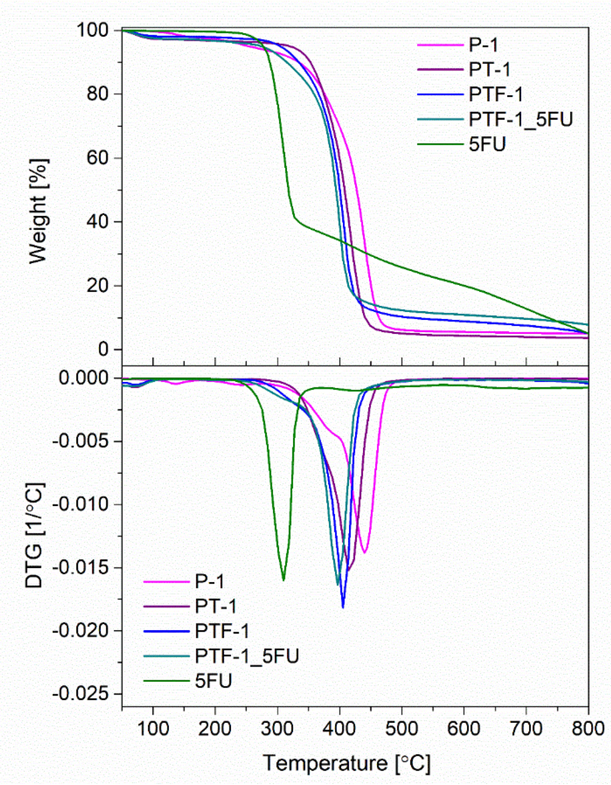

Figure S8. TG curves (top panel) and DTG curves (bottom panel) of **P-1**, **PT-1**, **PTF-1**, **PTF-1** with 5-FU, and 5-FU.

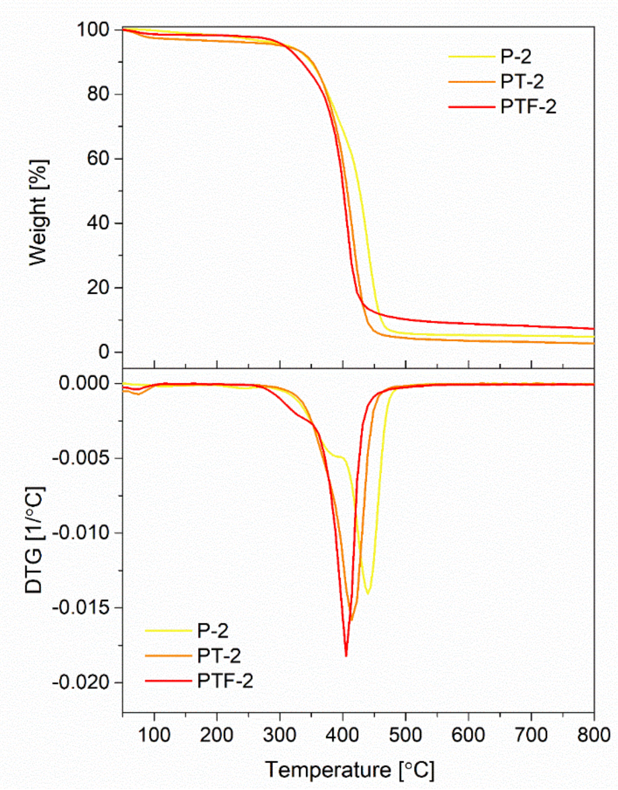

Figure S9. TG curves (top panel) and DTG curves (bottom panel) of **P-2**, **PT-2**, and **PTF-2**.

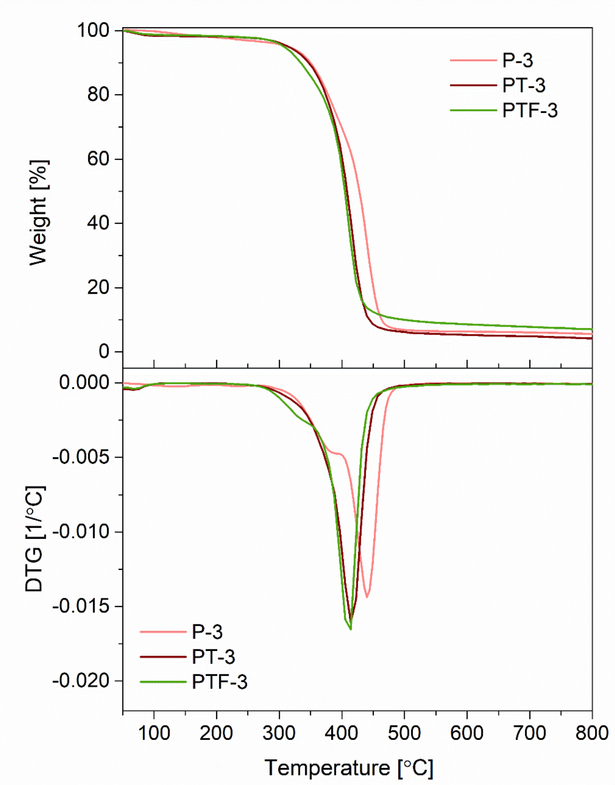

Figure S10. TG curves (top panel) and DTG curves (bottom panel) of **P-3**, **PT-3**, and **PTF-3**.

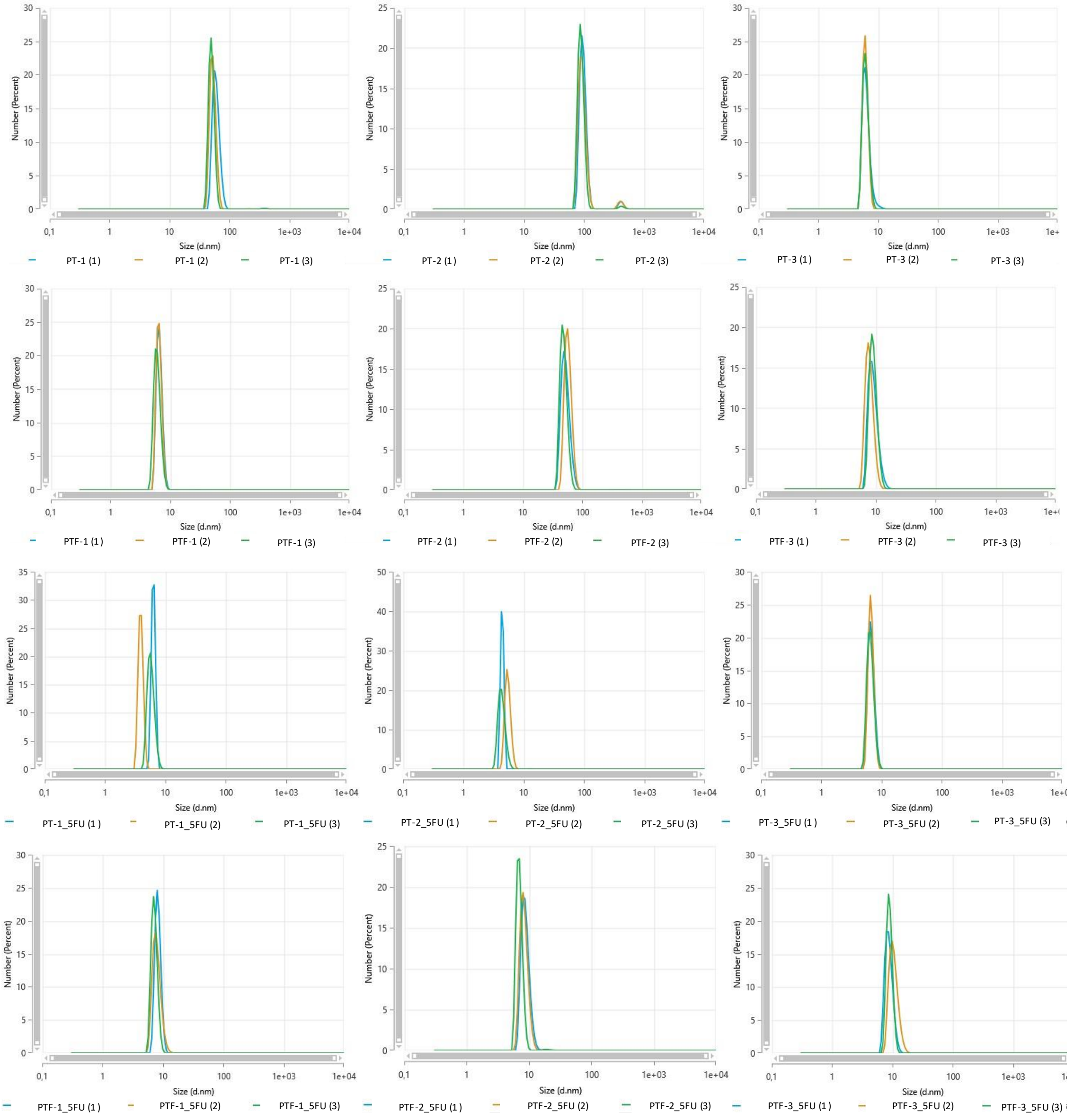

Figure S11. MADLS (size by number) measurements data of prepared polymers, **PT-(1-3)** and **PTF-(1-3)**, with or without presence of 5-FU.

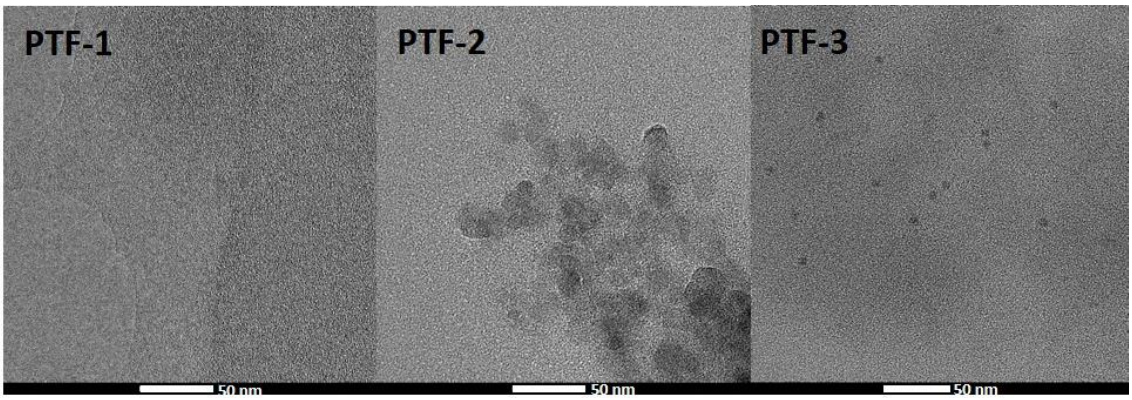

Figure S12. TEM images of polymers modified with folic acid.
