# Supplementary figures and images for: Folic-Acid-Conjugated Thermoresponsive Polymeric Particles for Targeted Delivery of 5-Fluorouracil to CRC Cells
Source: Int J Mol Sci. 2023 Jan 10;24(2):1364. doi: 10.3390/ijms24021364 (PMC9861804; doi:10.3390/ijms24021364)

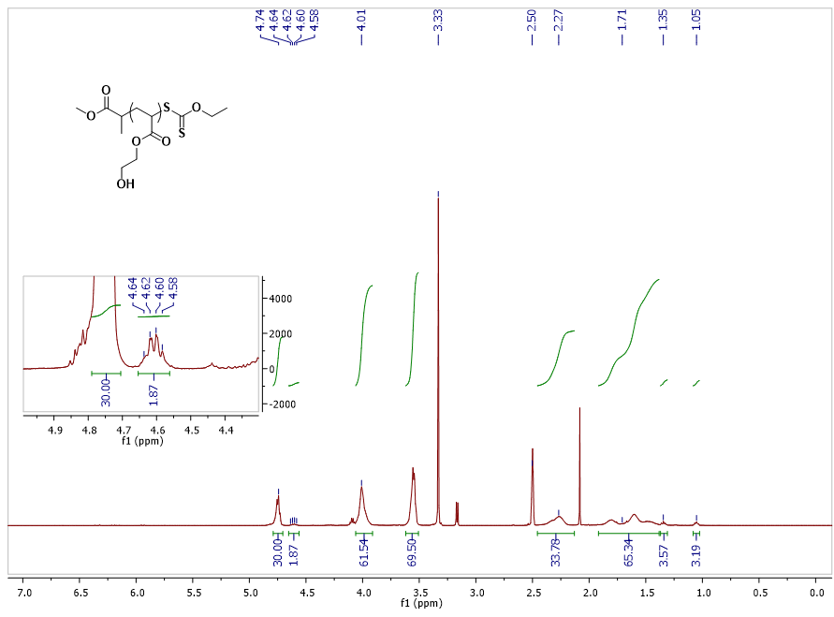

Supplement: Supplementary file 1 [file ijms-24-01364-s001.zip › Figure S1.tif]

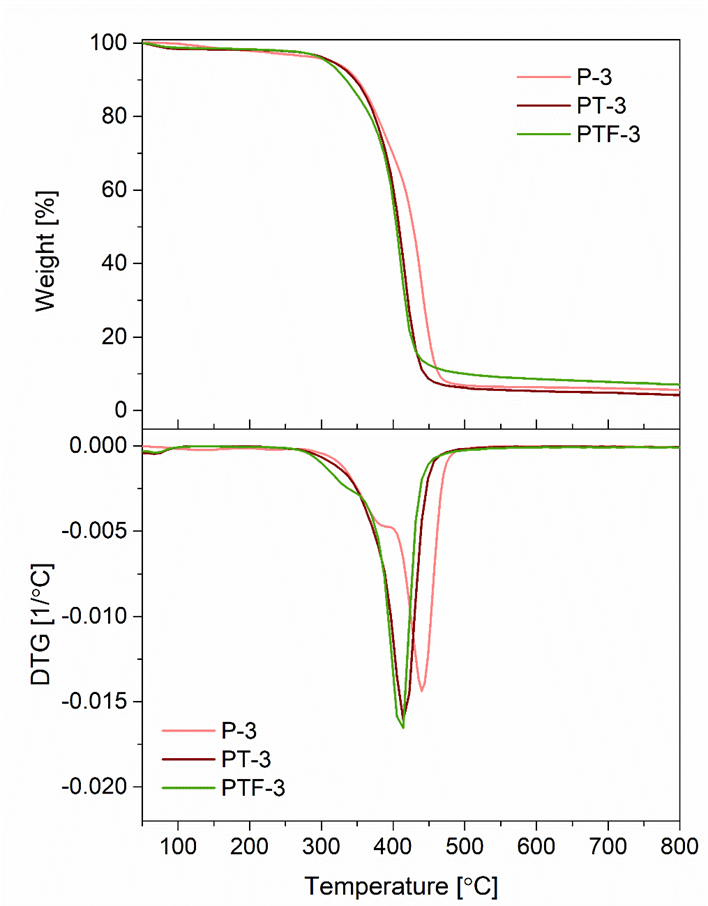

Supplement: Supplementary file 1 [file ijms-24-01364-s001.zip › Figure S10.tif]

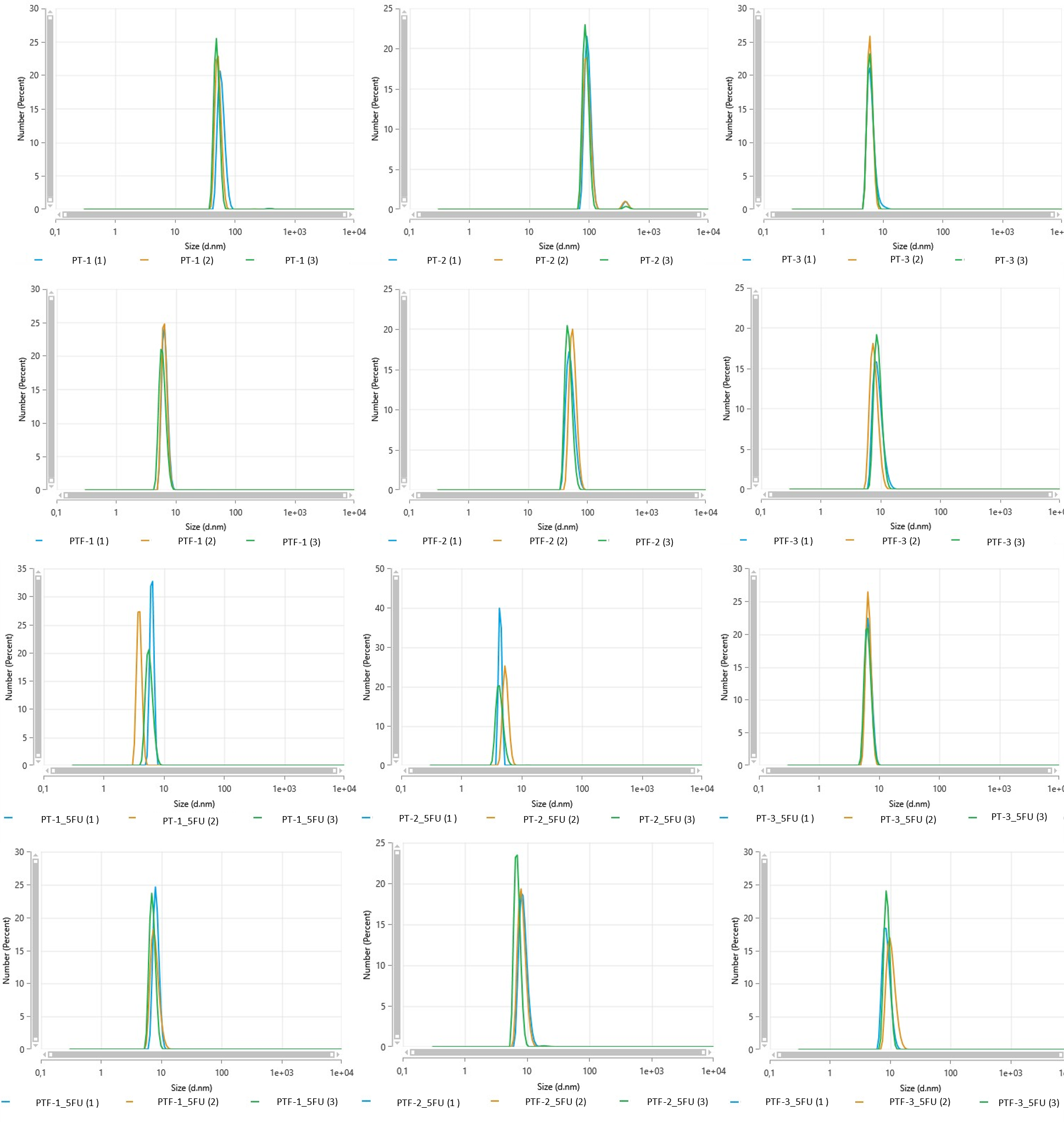

Supplement: Supplementary file 1 [file ijms-24-01364-s001.zip › Figure S11.tif]

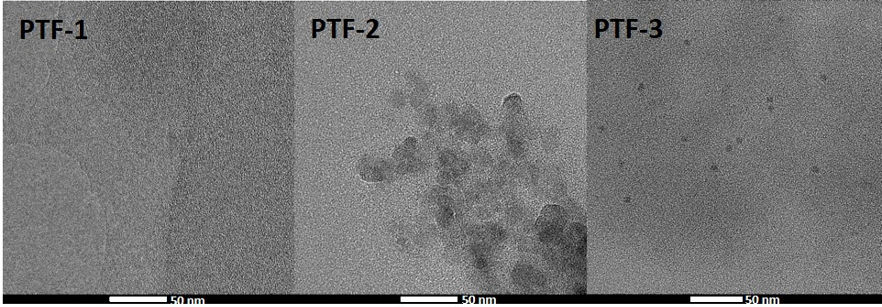

Supplement: Supplementary file 1 [file ijms-24-01364-s001.zip › Figure S12.tif]

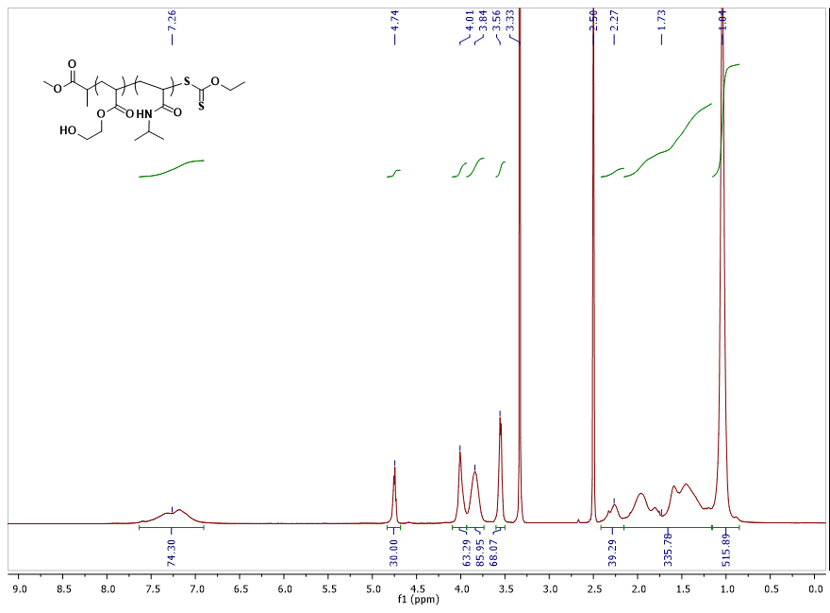

Supplement: Supplementary file 1 [file ijms-24-01364-s001.zip › Figure S2.tif]

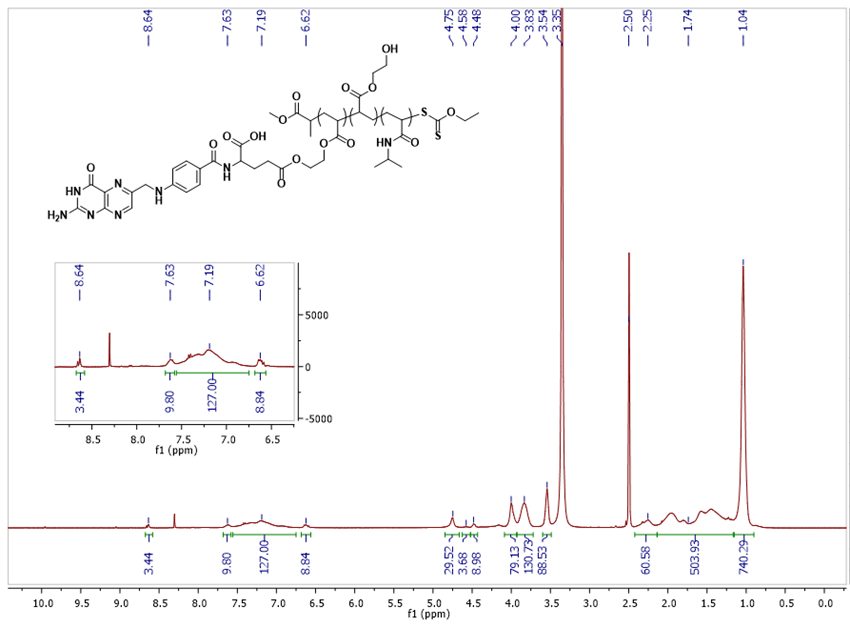

Supplement: Supplementary file 1 [file ijms-24-01364-s001.zip › Figure S3.tif]

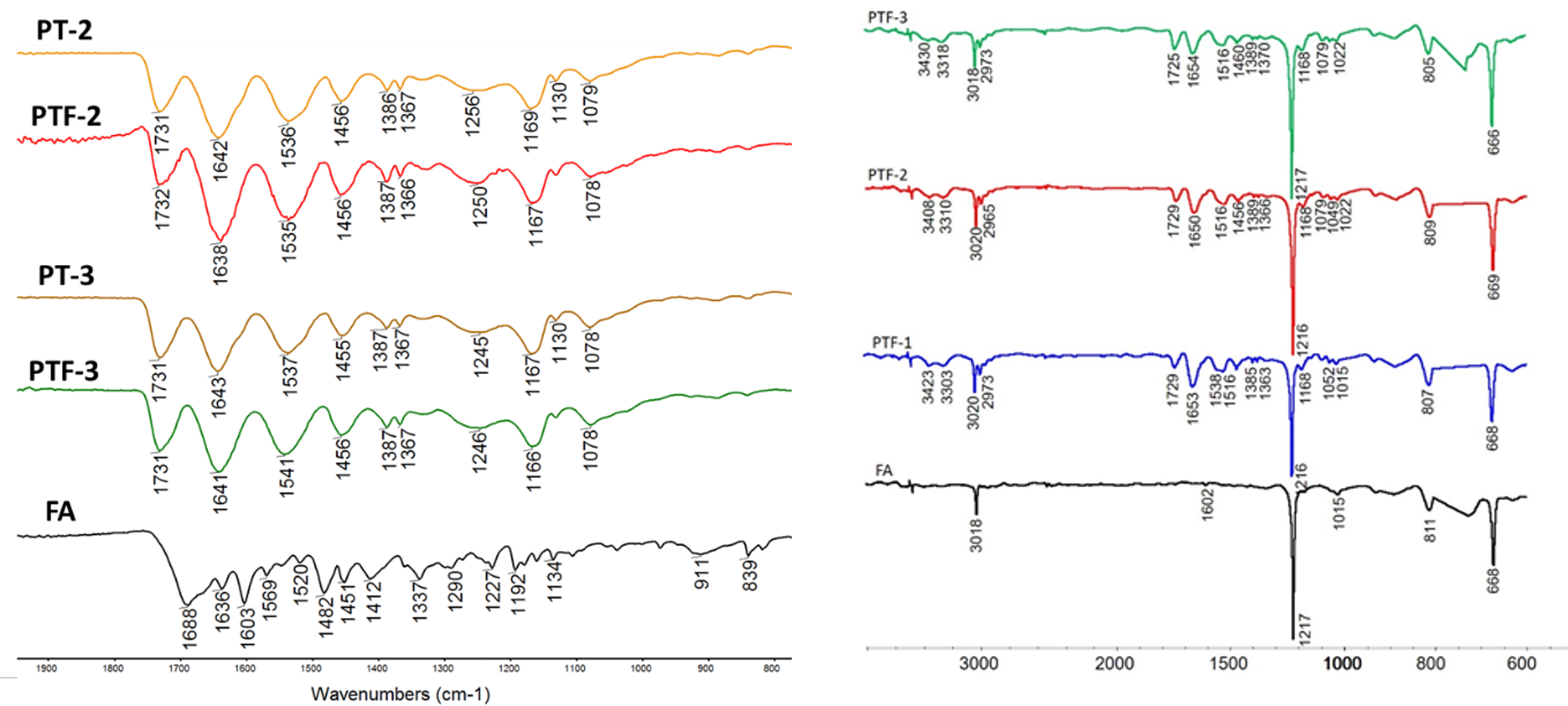

Supplement: Supplementary file 1 [file ijms-24-01364-s001.zip › Figure S4.tif]

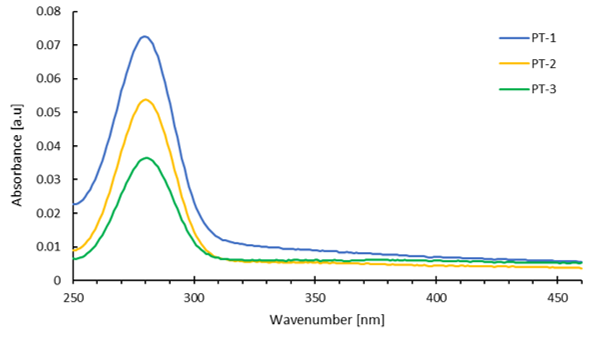

Supplement: Supplementary file 1 [file ijms-24-01364-s001.zip › Figure S5.tif]

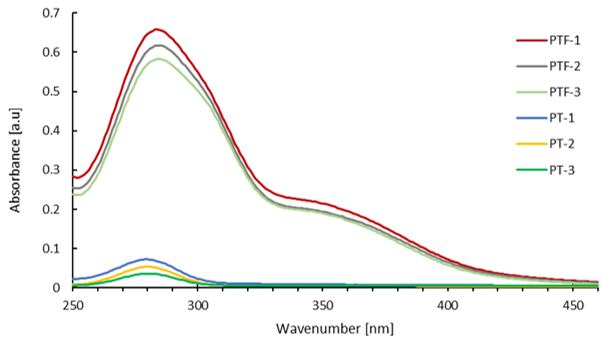

Supplement: Supplementary file 1 [file ijms-24-01364-s001.zip › Figure S6.tif]

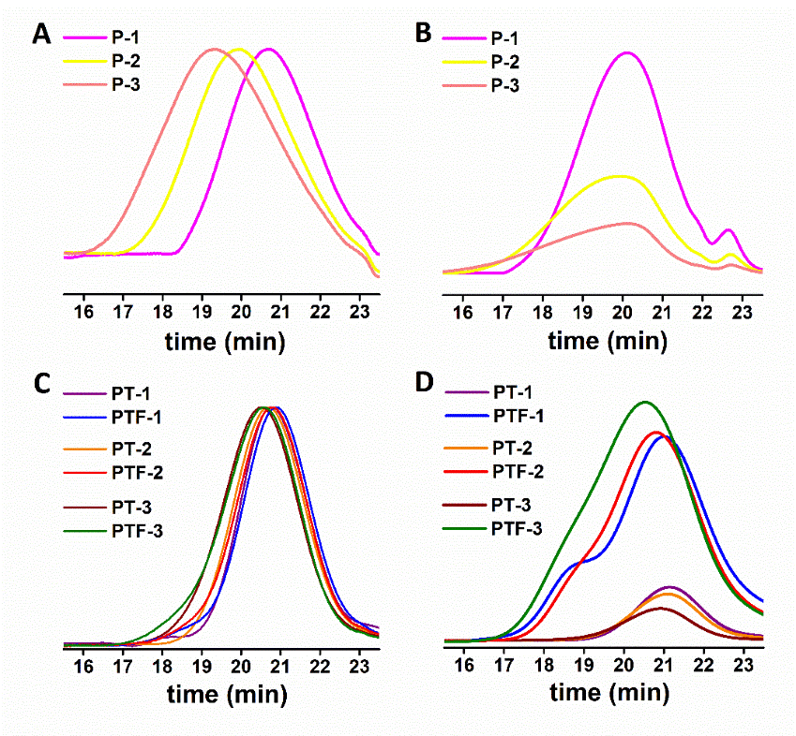

Supplement: Supplementary file 1 [file ijms-24-01364-s001.zip › Figure S7.tif]

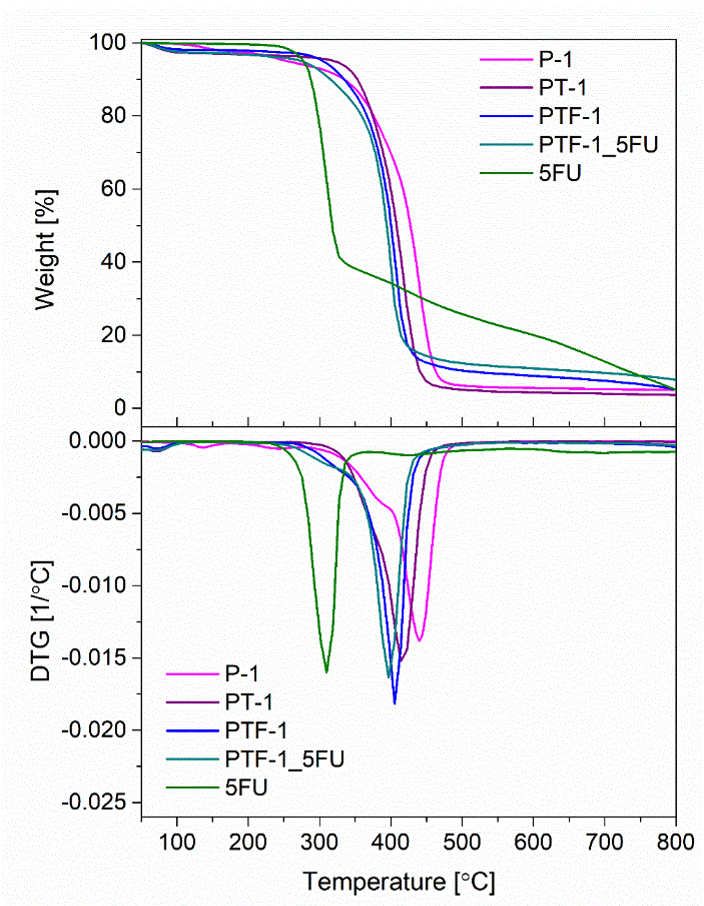

Supplement: Supplementary file 1 [file ijms-24-01364-s001.zip › Figure S8.tif]

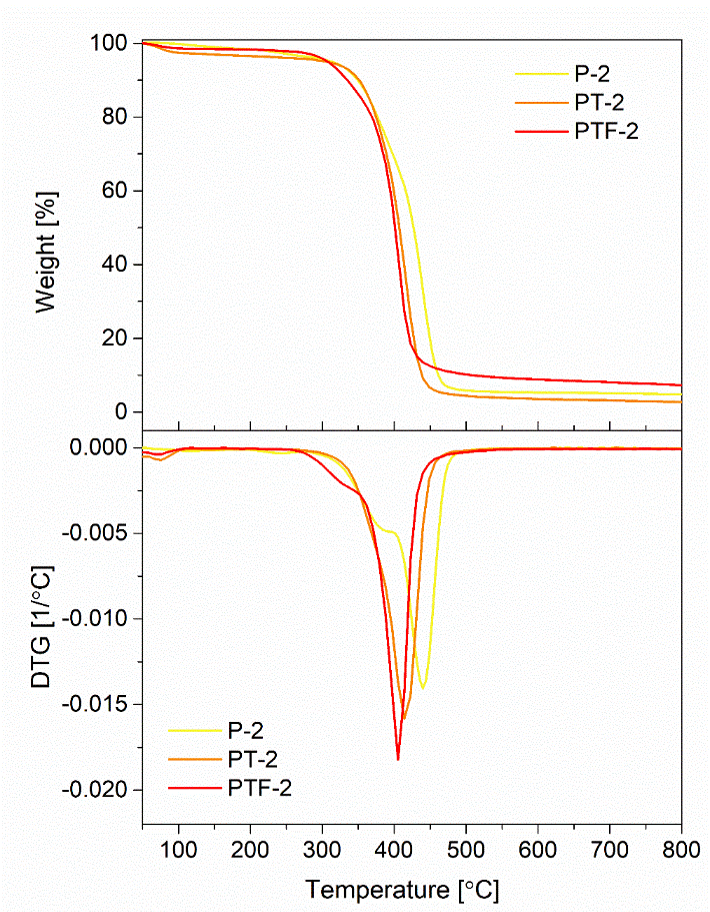

Supplement: Supplementary file 1 [file ijms-24-01364-s001.zip › Figure S9.tif]
